# Observation of polaritonic flat-band bound states in the continuum in a 2D magnet

**DOI:** 10.1038/s41467-026-76785-w

**Published:** 2026-08-19

**Authors:** Fuhuan Shen, Jiahao Ren, Zhiyi Yuan, Kai Wu, Sai Yan, Kunal Prasad, Hai Son Nguyen, Rui Su

**Affiliations:** 1 Division of Physics and Applied Physics, School of Physical and Mathematical Sciences, Nanyang Technological University, Singapore, Singapore; 2 CNRS-International-NTU-Thales Research Alliance (CINTRA), IRL 3288, Singapore, Singapore; 3 School of Electrical and Electronic Engineering, Nanyang Technological University, Singapore, Singapore; 4 Ecole Centrale de Lyon, INSA Lyon, Université Claude Bernard Lyon 1, CPE Lyon, CNRS, INL, UMR5270, Ecully, France

**Keywords:** Two-dimensional materials, Polaritons, Nanophotonics and plasmonics

## Abstract

Flat-band bound states in the continuum (BICs) are topological states with suppressed group velocity and robustness against radiation loss, offering a powerful platform for the exploration of non-Hermitian, nonlinear, topological phenomena and device applications. Van der Waals (vdW) metasurfaces have recently emerged as promising candidates for sustaining BICs and hybridizing with material transitions. However, the realization of flat-band BICs remains elusive. Here, we experimentally demonstrate high-order polaritonic BICs on a wide-angle flat band utilizing a subwavelength (~${\lambda }_{0}$ /35) metasurface based on a vdW magnet CrSBr. The large oscillator strength of direct excitons in CrSBr enables near-ultrastrong coupling with the photonic BIC-band, leading to a polaritonic band with strongly suppressed angular dispersions. Remarkably, the second-order polaritonic BIC-band becomes flattened, with a vanishing energy variation over a wide angular range. In addition, we find that these polaritonic BIC-bands vanish in the transverse magnetic configuration, while leading to hyperbolic exciton-polaritons within the Reststrahlen band. Our findings underscore CrSBr as an exceptional platform for exploring flat-band photonics and polaritonics, paving a new avenue for advances in next-generation optical and quantum technologies.

## Abstract (web summary)

Flat-band bound states in the continuum are localized optical modes with near-zero group velocity, and in their idealized form, infinite Q-factors. Realization of such states is challenging, but here, Shen and coauthors demonstrate polaritonic bound states in the continuum in a metasurface based on the van der Waals magnet, CrSBr.

## Introduction

Photonic bound states in the continuum (BICs) are perfectly localized optical modes that exhibit no radiation leakage to the environment while coexisting with extended waves of the same energy^1–3^. Their unique properties, including inherent topological characteristics^2^ and infinite *Q* factors^4,5^, drive advances in nanophotonics and light-matter interactions^6^, enabling applications in nonlinear optics^4,7–11^ and chiral sources^12–14^. However, deviations from symmetric geometry or normal excitation often lead to significant degradation of *Q* factors and energy shifts in conventional BICs^5,15,16^. These challenges can be effectively addressed by the concept of flat-band BICs, which feature near-zero group velocity and robustness against radiation loss^17–22^. Nonetheless, previous demonstrations have typically utilized passive media with band edge coupling^19,20^ or Moiré stacking^17,18^, which either limit the momentum range for flat bands or increase the modal volume and working wavelengths due to the enlarged supercell units. In this context, achieving flat-band BICs with strong light-matter coupling remains highly challenging.

In parallel, Van der Waals (vdW) materials have emerged as promising building blocks for dielectric nanophotonics due to their high refractive index, wide transparency range, and intrinsic excitons, in contrast to traditional semiconductors like silicon^23–30^. As a notable example, BIC-exciton-polaritons, hybrid states of photonic BICs and excitons, have recently been demonstrated in bulk vdW transition metal dichalcogenides (TMDCs) metasurfaces^31,32^. The hybridization of light and matter flattens the highly dispersive BIC-band into a more excitonic-like dispersion. Nevertheless, the bands remain far from true flat bands and can be strongly dampened by the intrinsic loss of excitons^31^. Moreover, bulk TMDCs typically exhibit low quantum efficiency due to their indirect bandgap nature^33^. Recently, the emerging vdW magnet CrSBr has shown promise as an exceptional candidate to tackle these challenges. Unlike TMDCs, it features anisotropic direct-bandgap excitons across all thicknesses^34–38^. Due to the magnetic ordering below the Néel temperature (T_N_ = 132 K), the excitonic oscillator strength is significantly enhanced (>1.5 (eV)^2^) along the magnetically easy axis (b-axis), accompanied by a simultaneously sharpened resonance linewidth (<1 meV). These unique characteristics enable the ultrastrong exciton-photon coupling regime even in a single CrSBr flake^34,35^,which distinguishes CrSBr from other vdW materials, highlighting its potential to strengthen BIC–exciton coupling and enable polaritonic flat-band BICs over a broad angular range. However, the experimental demonstration remains elusive.

Here, we experimentally demonstrate high-order polaritonic BICs on a wide-angle flat band, denoted as “polaritonic flat-band BICs”, based on vdW magnet CrSBr metasurfaces. By patterning CrSBr flakes into nanograting structures, we achieve the near-ultrastrong coupling regime between photonic BICs and the intrinsic excitons in CrSBr under the transverse electric (TE) configuration. The dominant excitonic contributions result in strongly suppressed angular dispersions of the polaritonic BIC-band. Notably, increasing the filling factor of the nanograting leads to the emergence of a second-order polaritonic BIC-band, exhibiting nearly vanishing dispersion (flat-band properties) and enhanced *Q* factor exceeding 1500. Under a transverse magnetic (TM) configuration, while all the polaritonic BIC-bands disappear, hyperbolic exciton-polaritons (HEPs) arise within the Reststrahlen (RS) band where the permittivity along the b-axis is negative. The HEPs exhibit angular-invariant dispersions in both reflectance and photoluminescence (PL) measurements, due to the strong subwavelength confinement of the electromagnetic field. Our findings highlight the unique capability of vdW magnet CrSBr to intrinsically tune the photonic properties of the metasurface via its excitons, opening new opportunities for advanced photonic and polaritonic devices in quantum circuits and magneto-optic applications.

## Results

### Suppressed angular dispersion of polaritonic BIC-band in CrSBr gratings

**Fig. 1:**
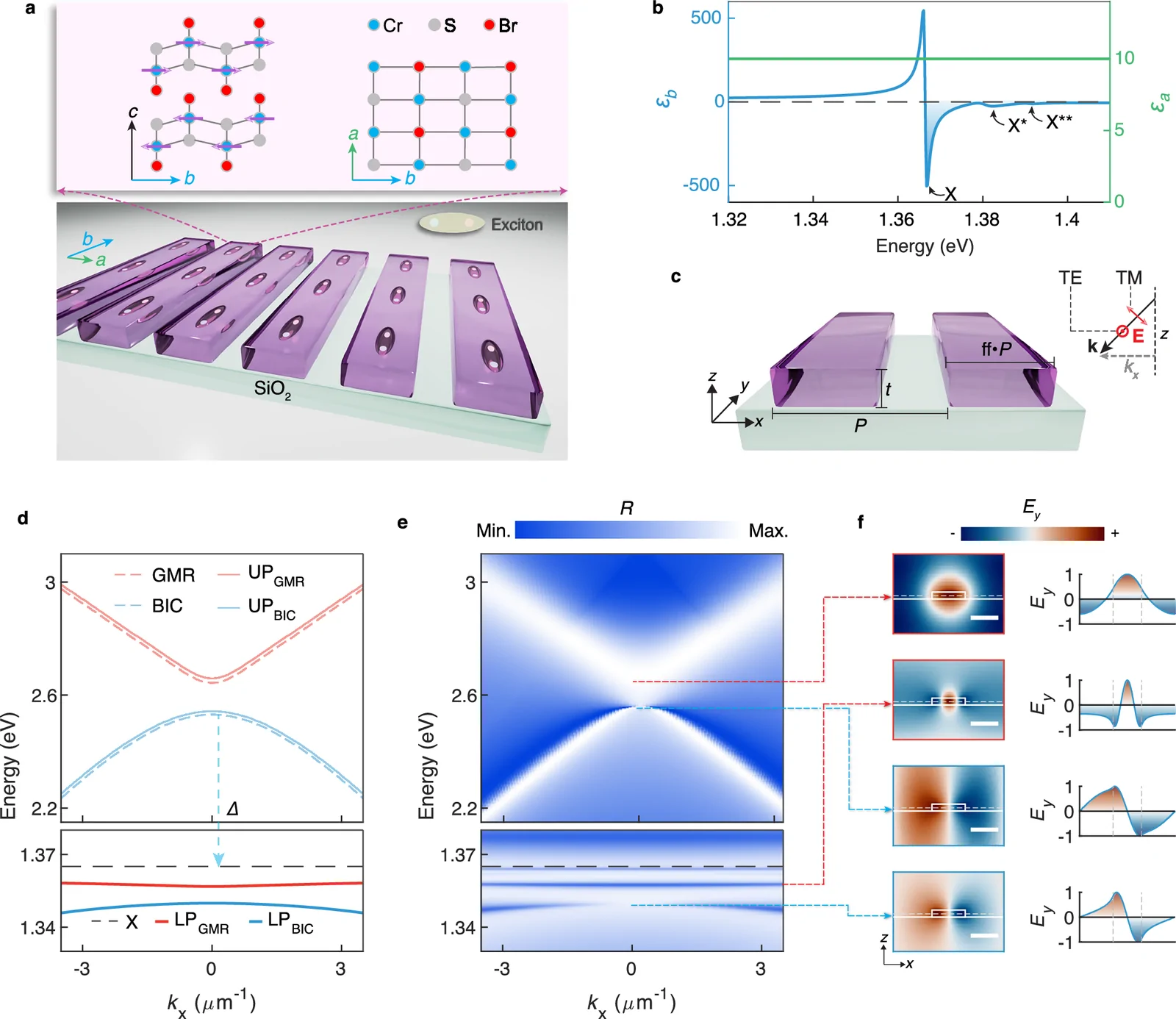
Suppressed angular dispersion of LP_BIC_ band in CrSBr nanogratings. **a** A schematic illustration of the CrSBr nanograting (bottom). The blue and red circles represent the electron and hole forming the exciton, respectively. The exciton is aligned with the b-axis. The top panel exhibits the atomic arrangement of CrSBr, with purple arrows indicating the magnetic moments. **b** Real parts of the permittivities of CrSBr along the b-axis (blue) and a-axis (green). Exciton X, and its sidebands X* and X** are noted by black arrows. **c** Definition of geometric parameters of the CrSBr grating: *P* denotes the period, *t* represents the thickness, and the filling factor (*ff*) is defined as the width *w* of the grating bar divided by *P*. The *y*-axis of CrSBr aligns with the long axis of the grating bar, while the *x*-axis is defined as the direction perpendicular to the long axis. The TE configuration is oriented with the electric field parallel to the long axis, whereas the TM configuration has the electric field within the incident plane and the magnetic field along the long axis of the grating bar. **d** Angular dispersions of uncoupled GMR and BIC bands, UP_GMR_, UP_BIC_, LP_GMR_, LP_BIC_, and exciton X, respectively. Δ represents the detuning between the uncoupled BIC mode and exciton X. **e** Calculated angle-resolved reflectance spectra of the CrSBr nanograting, which correspond to (**d**). **f** Left panel: near-field (${E}_{y}$) distributions of UP_GMR_, LP_GMR_, UP_BIC_, and LP_BIC_, respectively, in an *x*–*z* profile. Right panel: corresponding amplitude distributions of ${E}_{y}$ at the middle of the grating bar. The gray dashed lines indicate the width of the grating bar. For the calculations in (**d**–**f**), P=400 ${\mbox{nm}}$, t=25 ${\mbox{nm}}$, and ${ff}=0.3$ were used.

CrSBr is a layered vdW material featuring an orthorhombic structure with space group *Pmmn* (D_2h_). Below the bulk Néel temperature (~132 K), CrSBr exhibits the A-type antiferromagnetic order with the magnetic moments oriented ferromagnetically along the b-axis within the ab-plane, while ordered antiferromagnetically along the c-axis (see top panel in Fig. 1a). The electronic and structural anisotropy, along with the magnet-correlated properties, result in the tightly bound quasi-1D excitons along the b-axis (magnetic easy axis)^37^. This behaviour is clearly reflected in the permittivities measured in different directions (Fig. 1b). Along the b-axis, the permittivity is dominated by the exciton X at around 1.3655 eV, showing a sharp resonance with a linewidth of around 0.85 meV. Additionally, we note the existence of sidebands above the energy of exciton X, labelled X* and X**^39^, which exhibit much lower oscillator strengths and broader linewidths compared to X (see “Methods”). In contrast, the permittivity along the a-axis (${\varepsilon }_{a}$) is effectively constant in the infrared range.

Here, the exfoliated CrSBr flakes, ranging from ~15–35 nm in thickness, are patterned into nanograting metasurfaces, as schematically illustrated in the bottom panel of Fig. 1a. TE modes are first studied where the electric field (**E**) is along the long axis (defined as *y*-axis) of grating bar (Fig. 1c). To couple the TE mode with excitons, b-axis of CrSBr also aligns with the *y*-axis, as schematically shown in the bottom panel of Fig. 1a. Under this TE configuration, the periodic structure (i.e., nanograting) introduces the diffractive coupling between two propagating waves within the CrSBr slab, resulting in the splitting of two photonic modes, with one being the bright mode, i.e., guided mode resonances (GMRs), and the other being the dark mode at the Γ point in Brillouin zone, namely BICs^40,41^ (also see Supplementary Section A). Correspondingly, the photonic bands sustaining these two modes will be denoted as the “photonic GMR-band” and “photonic BIC-band”, respectively.

Without excitons, the photonic GMR- and BIC-bands in our simulation results (dashed curves in Fig. 1d) exhibit parabolic dispersions with a photonic bandgap around the Γ point. These bands appear significantly above the energy of the original exciton X, with the detuning Δ between the energy of BICs and exciton X exceeding 1 eV. When excitons are included in the permittivity of CrSBr, the photonic GMR-/BIC-band independently hybridizes with the excitons, resulting in the formation of upper (UP_GMR_/UP_BIC_) and lower (LP_GMR_/LP_BIC_) polaritonic GMR-/BIC-bands, which can be described by the following Hamiltonian:

1 $$H={H}_{{cav}}+{H}_{{exc}}+g\left(\hat{a}{\sigma }_{+}+{\hat{a}}^{{{{\boldsymbol{{{\dagger}}} }}}}{\sigma }_{+}+h.c.\right)+\frac{{g}^{2}}{{\omega }_{a}}{({\hat{a}}^{{{{\boldsymbol{{{\dagger}}} }}}}+\hat{a})}^{2}$$

The first two terms ${H}_{{cav}}={\omega }_{c}(\frac{1}{2}+{\hat{a}}^{{{{\boldsymbol{{{\dagger}}} }}}}\hat{a})$ and ${H}_{{exc}}={\omega }_{a}(\frac{1}{2}+{\sigma }_{z})$ represent the uncoupled photonic modes (i.e., GMR or BIC modes) and excitons, and the third and fourth terms indicate the interaction part^42^. As the system approaches the ultrastrong coupling regime (see Supplementary Figs. S2 and S3 where the coupling strength *g* is close to $0.1{\omega }_{a}$), the fast-rotation term (${\hat{a}}^{{{{\boldsymbol{{{\dagger}}} }}}}{\sigma }_{+}$) will be kept and the so-called A^2^ term arises as the last term in Hamiltonian^42–44^. Due to the large detuning ($\Delta={{\omega }_{c}-\omega }_{a}$), the resulting UP bands (solid curves in the upper panel of Fig. 1d) with dominant photonic weight maintain the similar angular dispersions of the original photonic modes, but with a slight blue shift. In contrast, the corresponding LP bands (solid curves in the lower panel of Fig. 1d) show significantly suppressed angular dispersion, owing to dominant excitonic weight (details can be found in Supplementary Section B).

The angle-resolved reflectance spectra calculated using rigorous coupled-wave analysis (RCWA) provide clearer insights into the distinct behaviors of polaritonic GMR- and BIC-bands (Fig. 1e). While the UP_GMR_ band is highly dispersive and exhibits a broad linewidth, the LP_GMR_ band displays exciton-like behavior, with its angular dispersion significantly suppressed and linewidth reduced, primarily due to the dominant contributions from excitons X. In contrast, the UP_BIC_ band manifests as a vanishing mode (true BICs) as it approaches the normal direction (at the Γ point), evolving into quasi-BICs with finite linewidth when the symmetry protection is lifted at finite in-plane momentum. Similar to LP_GMR_, the LP_BIC_ band shows a reduced linewidth and greatly suppressed angular dispersion compared to its upper-branch counterpart (the UP_BIC_ band).

The corresponding calculated near-field distributions (Fig. 1f) reveal different features of GMR- and BIC-polaritons, respectively. GMR-polaritons exhibit symmetric near-field distributions (as shown in the first two images of Fig. 1f), enabling their coupling with far-field radiation. In stark contrast, BIC-polaritons exhibit antisymmetric field distributions, preventing their coupling with the radiative continuum due to the parity mismatch. Moreover, lower polariton branches exhibit greater field confinement than upper ones, as evidenced by the field distribution profiles at the center of grating bars (right panels of Fig. 1f), where the dashed lines indicate the boundary of CrSBr.

### Experimental demonstration of polaritonic BICs in CrSBr nanogratings

**Fig. 2:**
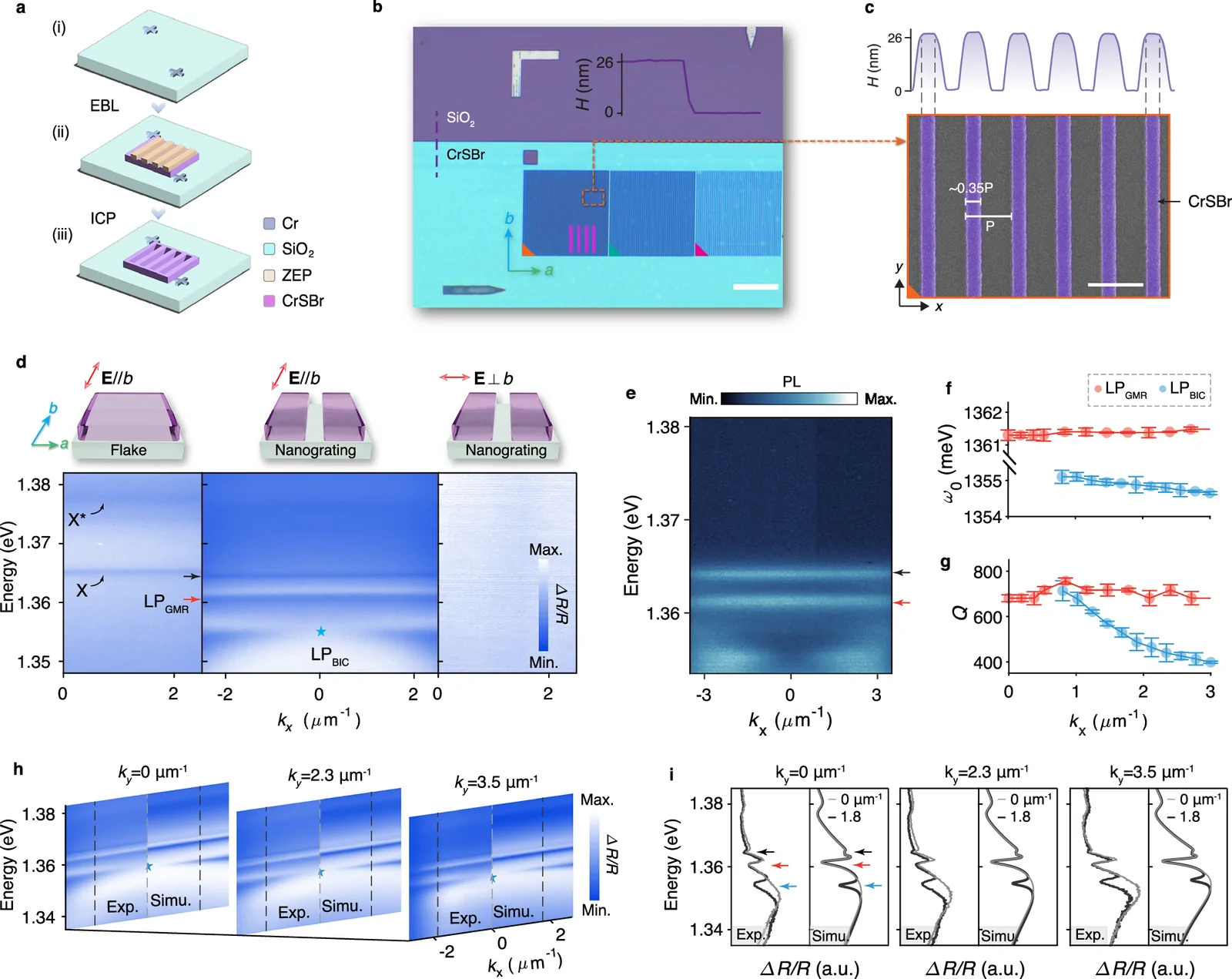
Experimental demonstration of BIC-polaritons in CrSBr nanogratings. **a** A schematic of the fabrication procedures for CrSBr nanogratings. **b** Microscopic image of CrSBr nanogratings. The inset shows the AFM height profile of the corresponding CrSBr flake indicated by the dashed purple line. The scale bar represents 10 μm, and the direction of the grating bars is indicated by a purple bar array. **c** Bottom panel: SEM image of a CrSBr grating with a filling factor of approximately 0.35. False color (purple) is used to emphasize the CrSBr grating bars. The scale bar represents 500 nm. Top panel: corresponding AFM height profile of the grating. **d** Angle-resolved reflectance spectra of bulk CrSBr flakes (left), the CrSBr nanograting with the excitation electric field along the b-axis (middle), and the CrSBr nanograting with the excitation electric field perpendicular to the b-axis (right). Black and red arrows indicate the assembly of high-order polaritonic bands and the first-order LP_GMR_, respectively. The blue star marks the vanishing signature at ${k}_{x}=0$ for LP_BIC_. **e** Corresponding angle-resolved PL for CrSBr nanograting. **f**, **g** Evolution of resonant frequencies (**f**) and *Q* factors (**g**) as a function of *k*_*x*_ for LP_GMR_ (red solid circles) and LP_BIC_ (blue solid circles), extracted through Lorentzian fittings from the data in (**e**). The error bars arise from the fitting process. **h** Measured and simulated angle-resolved reflectance spectra along the *k*_*x*_ direction with fixed ${k}_{y}$ values of 0, 2.3, and 3.5 μm^−1^, respectively. Gray and black dashed lines indicate the fixed momentum at ${k}_{x}=0$ and 1.8 μm^−1^, respectively. **i** Extracted spectra at ${k}_{x}=0$ (gray curves) and 1.8 μm^−1^ (black curves), corresponding to the data in (**h**). Unless otherwise specified, all tests are conducted at a temperature of 4 K.

To fabricate the CrSBr nanograting structures (Fig. 2a), the CrSBr flake is first exfoliated onto a PDMS substrate and then transferred onto a SiO_2_ (300 nm)/Si substrate that has been previously evaporated with Cr markers for locating the materials. This is then followed by a standard electron-beam lithography (EBL) process to create the top-layer nanograting pattern made of ZEP photoresist. Finally, using the ZEP nanopattern as the mask, the CrSBr flake is etched by the inductively coupled plasma (ICP) dry etching process. The ZEP residue is subsequently removed by the O_2_/Ar plasma (see more details in “Methods”). Compared to previous works^31,45^, the simplified fabrication procedures adopted here do not require a hard mask made of metal or silicon nitride, reducing residues on the CrSBr surface. Figure 2b exhibits the microscopic image of a representative CrSBr sample with a thickness of around 26 nm. The periods of these three gratings are fixed at 400 nm, but with various filling factors (defined by the width of the grating bar divided by the period, as schematically shown in Fig. 1c). The scanning electron microscope (SEM) image (bottom image of Fig. 2c) of one representative grating shows a filling factor of around 0.35. The corresponding atomic force microscopy (AFM) profile shows the same thickness of grating bars as that of the pristine flake, confirming the complete etching of the grating structure.

The angle-resolved differential reflectance spectra demonstrate completely distinct optical behavior of the CrSBr nanograting compared to the pristine CrSBr flake (Fig. 2d). As shown in the left panel of Fig. 2d, in pristine CrSBr flakes, only exciton X and its sideband X^*^ appear^34,35^. In contrast, in the CrSBr nanograting (middle panel), LP_GMR_ (marked by a red arrow) and LP_BIC_ bands (marked by a blue star at the Γ point which indicates the BICs) emerge, which is consistent with the simulated results in Fig. 1e. Notably, an additional polaritonic band, marked by a black arrow, appears below the energy of exciton X with a small but unambiguous red shift. It corresponds to an assembly of multiple higher-order lower polaritonic bands, which will be discussed later. All of the polaritonic bands disappear when the electric field is oriented perpendicular to the b-axis (right panel of Fig. 2d), due to the quasi-1D nature of excitons.

These polaritonic GMR- and BIC-bands are also evident in the PL measurements (Fig. 2e). By fitting the data in Fig. 2e, we further analyze the properties of these polaritonic bands. The extracted resonant frequency ${\omega }_{0}$ of LP_GMR_ band (Fig. 2f) is nearly independent of the in-plane momentum *k*_*x*_, while ${\omega }_{0}$ of LP_BIC_ band shows a slightly red shift (~ 1 meV) as *k*_*x*_ increases from 1–3 µm^−1^. The *Q* factor (Fig. 2g) of the LP_GMR_ band approaches 700 at the Γ point and shows a slight increase with the increasing *k*_*x*_. In contrast, the *Q* factor (attributed to the quasi-BIC modes at finite in-plane momentum) of the LP_BIC_ band significantly decreases from around 700 at *k*_*x*_ = 1 µm^−1^ to approximately 400 at *k*_*x*_ = 3 µm^−1^ due to the increased asymmetry as *k*_*x*_ increases.

In addition, we measured the angle-resolved reflectance spectra at different *k*_*y*_ values to demonstrate the dispersions of LPs across the entire in-plane momentum space (Fig. 2h). We observe that the resonant frequency and linewidth of LP_GMR_ and LP_BIC_ bands remain nearly invariant for different *k*_*y*_ values, which is more clearly reflected in the extracted spectra (Fig. 2i) at *k*_*x*_ = 0 and 1.8 µm^−1^, respectively. Moreover, the optical response vanishes at *k*_*x*_ = 0 µm^−1^ in the LP_BIC_ band (Fig. 2i), which serves as a hallmark of BICs.

### Observation of high-order polaritonic BICs on a flat band

**Fig. 3:**
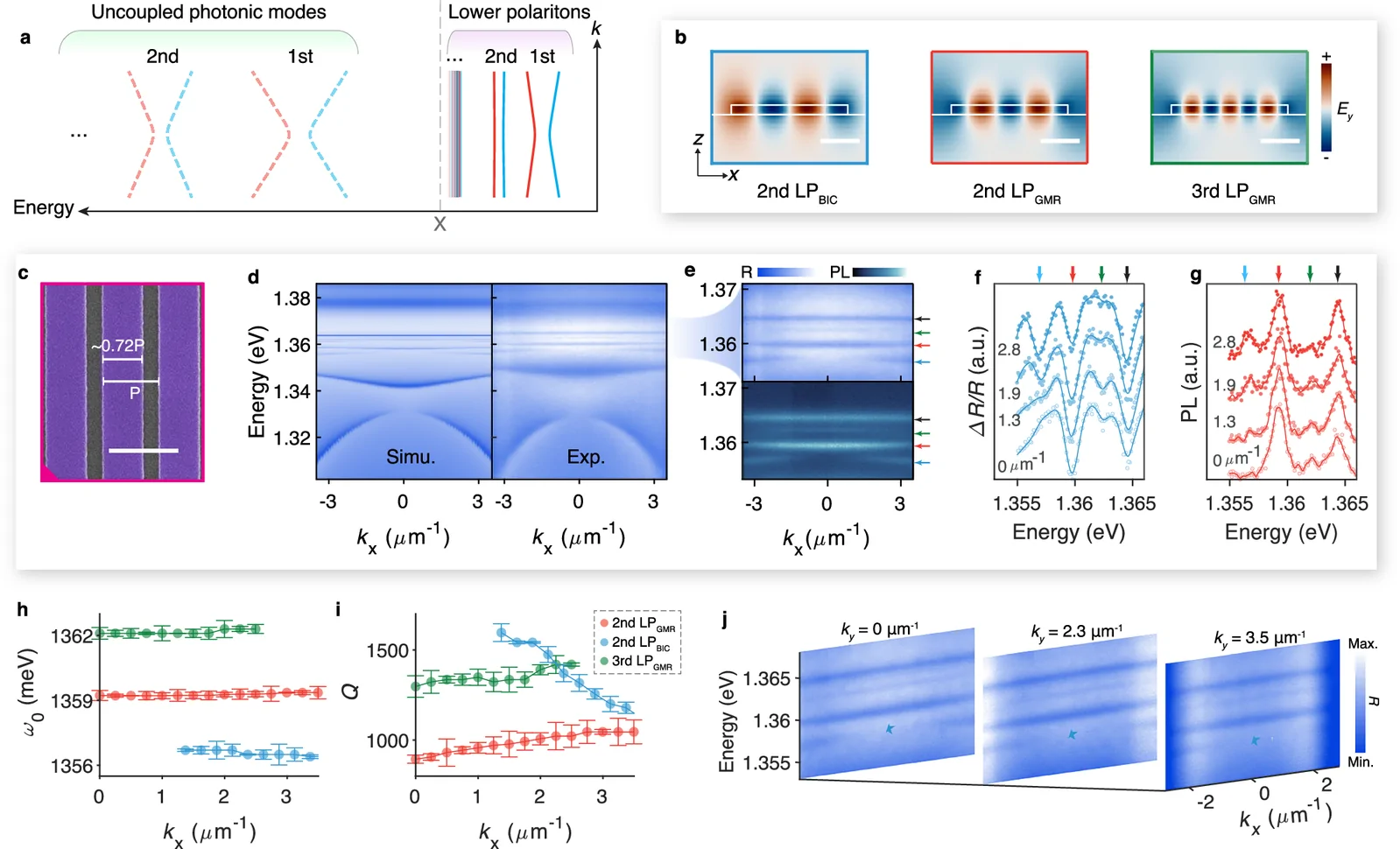
High-order polaritonic BIC- and GMR-bands. **a** Schematic illustration of the formation of high-order LP_BIC_ and LP_GMR_ bands. **b** Calculated near-field ${E}_{y}$ distributions for the second-order LP_GMR_, LP_BIC_, and third-order LP_GMR_ modes, respectively. **c** SEM image of the CrSBr grating with a filling factor of approximately 0.72. **d** Simulated (left) and measured (right) angle-resolved reflectance spectra of the grating shown in (**c**). **e** Zoomed-in angle-resolved reflectance and PL spectra corresponding to (**d**). Blue, red, green, and black arrows are adopted to indicate positions of second-order LP_BIC_, second-order LP_GMR_, third-order LP_GMR_, and assembly of higher-order modes, respectively. **f**, **g** Extracted reflectance (**f**) and PL (**g**) spectra at ${k}_{x}=0$, 1.3, 1.9, and 2.8 μm^−1^, respectively. The corresponding data are extracted from (**e**). **h**, **i** Extracted resonant frequencies ${\omega }_{0}$ (**h**) and *Q* factors (**i**) of the second-order LP_GMR_ (red circles), second-order LP_BIC_ (blue circles), and third-order LP_GMR_ (green circles), obtained by fitting the PL data in (**e**). The error bars arise from the fitting process. **j** Angle-resolved reflectance spectra along the *k*_*x*_ direction with ${k}_{y}$ values fixed at 0, 2.3, and 3.5 μm^−1^, respectively.

As aforementioned, in addition to the first-order LP_GMR_ and LP_BIC_ bands, there also exists a band appearing slightly below the energy of exciton X (Fig. 2d**)**, which is ascribed to the assembly of higher-order polaritonic bands. The formation of these polaritonic bands is schematically illustrated in Fig. 3a. The photonic GMR- and BIC-bands (represented by dashed curves) of *n*-th order (*n *= 1, 2, 3 …) appear sequentially above the energy of exciton X, corresponding to the folding and coupling of the same TE guided modes but operating at wavevector $\pm n\frac{2\pi }{P}$. Their hybridizations with exciton X result in LP bands with different orders, whose resonant energies approach that of exciton X with the increasing order number. In Fig. 2d, e, these higher-order polaritonic bands are too close in energy to be distinguished in the spectra, appearing within the same band.

However, through tuning the geometric parameters such as period or filling factor (see Supplementary Fig. S5), some higher-order polaritonic bands, such as the second- and third-order LP bands, can be effectively distinguished. When the filling factor is increased to 0.5 (noted by the green triangle in Fig. 2b and also in Supplementary Fig. S6a), the first-order LP_BIC_ and LP_GMR_ bands show unambiguous redshifts and become more angularly dispersive due to the increased photonic contributions in the resulting polaritons (Supplementary Fig. S8), particularly for LP_BIC_. More interestingly, an additional pair of LP_BIC_ and LP_GMR_ bands emerges between the first-order polaritons and exciton X (Supplementary Fig. S6b, c), serving as the second-order LP bands. As the filling factor is further increased to ~0.72 (as the SEM image in Fig. 3c shows**)**, the first-order LP_BIC_ and LP_GMR_ bands redshift and become more angularly dispersive (Fig. 3d). The zoom-in reflectance and PL spectra (Fig. 3e) unambiguously exhibit the dispersions of second-order LP_BIC_ (noted by a blue arrow), second-order LP_GMR_ (noted by a red arrow), and third-order LP_GMR_ (noted by a green arrow) bands. Compared to the second-order LP_GMR_ band, whose optical response is almost constant, the second-order LP_BIC_ band exhibits the transition from BIC mode to quasi-BIC mode when the symmetry is broken at finite *k*_*x*_. This is reflected more evidently in the corresponding reflectance (Fig. 3f) and PL (Fig. 3g) spectra at fixed *k*_*x*_ = 0, 1.3, 1.9, and 2.8 µm^−1^, respectively. The originally dark modes (true BICs) are brightened with the increasing ${k}_{x}$, showing increasingly pronounced dips or peaks. Additionally, to confirm their respective high-order nature, the corresponding near-field distributions at the Γ point are calculated (Fig. 3b). The node (i.e., *E*_*y*_ = 0) number increases from 2 for the first-order LP_GMR_ to 4 and 6 for second- and third-order LP_GMR_, respectively. For second-order LP_BIC_, the node number increases from 1 (first-order LP_BIC_) to 3. We noted that although in simulated results (left panel in Fig. 3d), the third-order LP_BIC_ band appears slightly below the third-order LP_GMR_ band, it is experimentally difficult to distinguish due to the narrow energy separation and low optical contrast.

Furthermore, we analyze the evolution of resonant frequencies and *Q* factors with *k*_*x*_ for these high-order polaritonic bands. Compared to the first-order polaritonic bands in Fig. 2f, the extracted resonant frequencies of second-order LP_GMR_ and LP_BIC_, and the third-order LP_GMR_ bands exhibit nearly vanishing angular dispersions (<0.2 meV∙µm), leading to polaritonic flat bands (Fig. 3h). The achievement of polaritonic flat bands benefits from the near-ultrastrong coupling regime arising from the large excitonic oscillator strength in CrSBr. This ultrastrong coupling regime enables exciton-like behaviour of LPs at large detuning (> 1 eV), while still maintaining sufficient separation to the bare exciton level.

Regarding the *Q* factors, similar to the first-order modes, high-order LP_BIC_ and LP_GMR_ bands exhibit opposite trends with the increasing *k*_*x*_ (Supplementary Section A). As shown in Fig. 3i, the *Q* factors of second- and third-order LP_GMR_ slightly increase as *k*_*x*_ increases, surpassing 1000 and 1300, respectively, when *k*_*x*_ reaches around 3 µm^−1^. In contrast, quasi-BIC modes in second-order LP_BIC_ band exhibit an unambiguous decrease as *k*_*x*_ increases, with its largest *Q* factor exceeding 1500 at *k*_*x*_ = 1.25 µm^−1^, which is, to the best of our knowledge, the record value for the vdW photonics in the near-infrared range^31^. This *Q* factor value is currently limited by the intrinsic *Q* factor (calculated by the exciton energy divided by its linewidth) of exciton X due to its dominant contributions to LP_BIC_. Increasing the contributions of photonic BIC-band to LPs, for example, by increasing the period or filling factor of gratings, is expected to further increase the total *Q* factor beyond the intrinsic loss by exciton. Moreover, due to its flat-band properties, the *Q* factors of the second-order LP_BIC_ band remain above 1000 even at *k*_*x*_ > 3 µm^−1^. Additionally, the angle-resolved reflectance spectra (Fig. 3j) at different *k*_*y*_ further confirm the flat-band properties of the second-order LP_BIC_ band across the entire in-plane momentum space. Similar flat-band behavior of the second-order LP_BIC_ band can also be observed in our CrSBr sample with a filling factor of around 0.5 (Supplementary Fig. S6g). Due to the limitation of collection angles in our experiment, the measured range of flat band only goes up to around 40 degrees, while our calculation indicates an almost 90-degree wide-angle flat band of high-order BIC-polariton branches (Supplementary Fig. S9).

### Hyperbolic exciton-polaritons under TM configuration

**Fig. 4:**
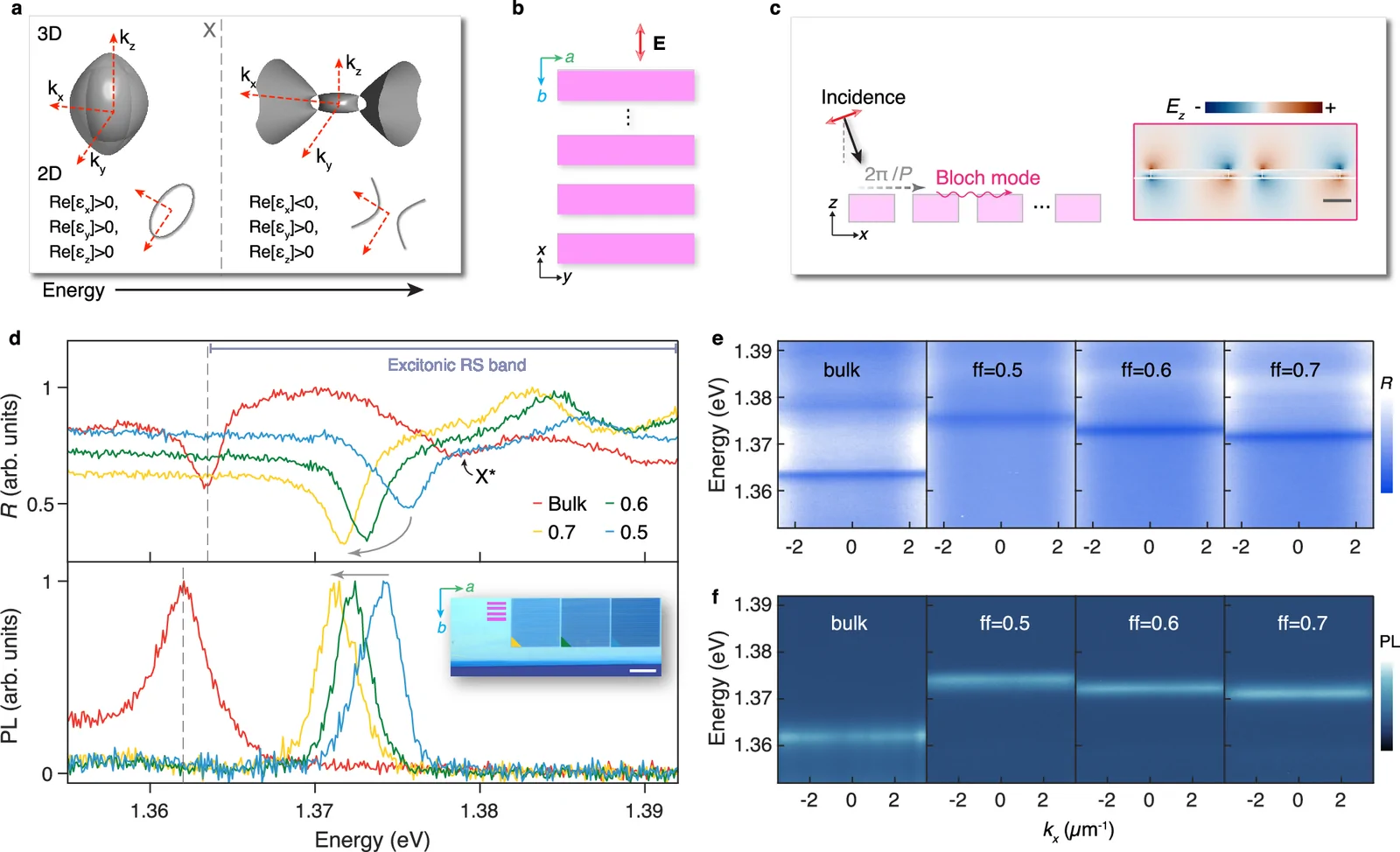
Hyperbolic exciton-polaritons in the excitonic RS band. **a** A schematic isofrequency surfaces (3D) and contours (2D) for Re[${\varepsilon }_{b}$] > *0* and Re[${\varepsilon }_{b}$] *< 0*, respectively, separated by the exciton resonance (gray dashed line). **b** Schematic illustration of the TM configuration of the CrSBr grating. The red double arrow indicates the projection of the electric field in the *xy*-plane. **c** Schematic illustration of the formation of hyperbolic exciton-polaritons (HEPs) (left) and calculated near-field distributions of HEPs (right). The scale bar represents 100 nm. **d** Normal reflectance (top panel) and PL spectra for bulk CrSBr (red curve) and gratings with varying filling factors: ${ff}=0.5$ (blue curve), ${ff}=0.6$ (green curve), and ${ff}$*=* 0.7 (yellow curve). The slight difference between the reflectance dip and PL peak of bulk CrSBr is attributed to the Stokes shift. Inset: microscope image of the CrSBr gratings under the TM configuration. Th**e** scale bar represents 10 μm. **e**, **f** Measur**e**d angle-resolved reflectance (**e**) and PL (**f**) spectra corresponding to the results in (**d**).

As indicated in Fig. 1b, CrSBr is an anisotropic material with distinct in-plane permittivities along different directions. Below the energy of exciton X, CrSBr is a usual elliptical material (i.e., Re[${\varepsilon }_{x}$]≠Re[${\varepsilon }_{y}$]≠Re[${\varepsilon }_{z}$] ˃ 0, here *x*-, *y*-, and *z*-axes correspond to b-, a-, and c-axes, respectively, as indicated in Fig. 4b) whose isofrequency surface consists of two closed surfaces (left part in Fig. 4a). This scenario corresponds to the above discussion under TE configuration. However, above the energy of exciton resonance, the large oscillator strength and the sharpened linewidth of exciton X drive the permittivity to be negative along the b-axis, yielding excitonic RS band^46^. Within this band, the isofrequency surface becomes highly nontrivial as shown in the right panel of Fig. 4a. An isofrequency surface with Re[${\varepsilon }_{y}$] ≠ Re[${\varepsilon }_{z}$] > 0 > Re[${\varepsilon }_{x}$] consists of a hyperboloid opening along the *k*_*x*_ direction^47^, thus enabling the generation of HEPs^39^. HEPs are intrinsically TM-polarized below the material light cone, showing large in-plane wavevectors, subdiffractional wavelength confinement, and enhanced light-matter interaction compared with conventional exciton-polaritons^39^. Usually, these HEPs are difficult to probe in the far field and can only be accessed through near-field spectroscopy, such as scattering-type scanning near-field optical microscopy (s-SNOM)^39^. Here, we overcome this limitation by employing a nanograting structure supplying an in-plane reciprocal lattice vector *G* = $\frac{2\pi }{P}$ (left panel of Fig. 4c), which enables phase matching between a finite-momentum bulk HEP^39^ and a radiative Bloch mode that can be excited and detected in the far field. Similar to probing the surface plasmon polaritons with a grating coupler^48^, the original propagating HEPs become Bloch modes by our periodic structures (as the near field distributions in Fig. 4c indicate).

To probe HEPs in experiments, our measurement is set under TM configuration where both the in-plane component of electric field and b-axis of CrSBr are aligned with the short axis of nanograting bar (as schematically shown in Fig. 4b). The period *P *= 400 nm (G~15.7 ${\mu m}^{-1}$) is chosen to match the momentum range of bulk HEP which is beyond the light cone of CrSBr (more details can be seen in Supplementary Section K). Our measured reflectance spectra at normal direction (Fig. 4d) exhibit HEPs lying in the energy range between the exciton X and sideband X* (Fig. 4d), and the corresponding PL measurements also show consistent results (Fig. 4d). With the increase of filling factor, we further observe an unambiguous redshift trend (as indicated by the gray arrows) of the HEP modes. This is because the larger filling factor increases the average permittivity of the CrSBr slab that shifts the Bloch-mode phase-matching condition for the finite-momentum bulk HEPs coupled out by the reciprocal lattice vector *G*. Additionally, our angle-resolved reflectance (Fig. 4e) and PL (Fig. 4f) spectra also exhibit flat-band behavior of these HEP modes. Although this resembles the nearly flat lower-polariton branches under TE configuration, the physical origin is different: the TE polarized lower polaritons are flattened by their dominant excitonic fraction, whereas the weak angular dependence of the HEP resonance is associated with the strongly subwavelength nature of HEPs in CrSBr and their grating-assisted outcoupling to the far field^22,39^. However, under TM configuration, we do not observe LP_BIC_ and LP_GMR_ bands, which emerge in other vdW grating structures^30,49^. Generally, the TM guided modes exhibit a thickness cutoff^30^,requiring field retardation along the *z*-direction (i.e., c-axis of CrSBr) to form a displacement current loop^50,51^. For the present thickness (<30 nm) and spectral range, the structure is below the cutoff for a TM guided mode, thereby preventing the formation of TM guided GMR- or BIC-modes.

## Discussion

Our work presents the first demonstration of polaritonic flat-band BICs in the deeply subwavelength CrSBr nanogratings. The angular dispersions of LP_BIC_ bands are significantly suppressed due to the ultra-strong coupling and large detuning between photonic BIC-bands and excitons in CrSBr. The increasing filling factor of the grating enables the observation of second-order LP_BIC_ band, which exhibits the exceptional robustness of resonant energies and *Q* factors against the inclined angle. The polaritonic flat-band BICs achieved through ultrastrong coupling in CrSBr nanogratings outperform the traditional dispersive BICs and the flat-band BICs achieved through the hybridization of different energy bands. In addition, we observe HEPs spectrally within the RS band under the TM configuration by angle-resolved reflectivity and PL measurements, showing flat-band dispersions.

Our findings highlight the significant potential of the vdW magnet CrSBr for advanced applications in nanophotonics and polaritonics. Recently, pioneering studies have explored the nonlinear properties and polariton condensates in CrSBr^52^. However, these efforts have been constrained in the bulky Fabry-Pérot cavity structures, which would hinder minimizations in device footprint and compatibility with on-chip applications for integrated photonics and quantum sources. The deliberate designs of CrSBr structures overcome these limits and allow for the giant flexibility to manipulate the novel optical properties such as light wavefronts and topological behaviors, paving the way for the interplay of the photonic, electronic, and magnetic properties in this promising platform.

## Methods

### Device fabrications

The pristine CrSBr flakes are prepared on PDMS by mechanical exfoliation from the CrSBr crystal bought from HQ graphene. The CrSBr flakes with homogeneous and target thickness (10–40 nm) are selected according to the microscope images. The SiO_2_ (300 nm)/Si substrates with markers are prepared by the standard EBL, Cr evaporation, and lift-off procedures. Then, using the dry-transfer method, the selected CrSBr flake is transferred onto the targeted area marked by the Cr markers. Next, the CrSBr flake is cleaned by the O_2_/Ar plasma to remove the residues on the surface and then is spin-coated with ZEP (pristine ZEP is diluted by the anisole by the volume ratio of 1:1) photoresist at 5000 rpm for 1 min. A standard EBL is applied and followed by a development process (immersed in pentyl acetate for 1 min at zero degrees and rinsed in isopropyl alcohol for 30 s at room temperature). Last, the CrSBr flake with top layer nanopatterns is etched by the ICP process at a pressure of 6 mTorr with the gas Cl_2_ (20 sccm)/Ar (5 sccm)/O_2_ (3 sccm) for 30 s. The HF/ICP power is set as 100 W/400 W, respectively. After ICP, the residual ZEP on the surface of CrSBr can be removed by the Ar/O_2_ plasma.

### Fitting parameters

The permittivity of CrSBr along the b-axis was extracted by fitting the measured reflection spectra of unpatterned CrSBr area with a multi-Lorentzian model:

2 $$H{\varepsilon }_{b}\left(\omega \right)={\varepsilon }_{b0}+{\sum}_{j}\frac{{f}_{j}}{{\omega }_{j}^{2}-{\omega }^{2}+i\omega {\gamma }_{j}}$$

where ${\varepsilon }_{b0}=11$ is the background permittivity, ${f}_{j}$, ${\omega }_{j}$, and ${\gamma }_{j}$ are the oscillator strength, resonant frequency, and linewidth of the exciton, respectively. The fitting parameters are listed below:

$${{{\rm{X}}}}:\;{f}_{1}=1.6{({{{\rm{eV}}}})}^{2},{\omega }_{1}=1.3655\;{{{\rm{eV}}}},\; {{{\rm{and}}}} \;\; {\gamma }_{1}=0.85{{{\rm{meV}}}}.$$

$${{{{\rm{X}}}}}^{*}:\;{f}_{2}=0.2\,{({{\mathrm{eV}}})}^{2},\,{\omega }_{2}=1.3801\,{{\mathrm{eV}}},\; {{\mathrm{and}}} \;\,{\gamma }_{2}=5\,{{\mathrm{meV}}}.$$

$${{{{\rm{X}}}}}_{2}:\;{f}_{4}=1.1\,{({{\mathrm{eV}}})}^{2},\,{\omega }_{4}=1.76\,{{\mathrm{eV}}},\; {{\mathrm{and}}} \;\,{\gamma }_{4}=23\,{{\mathrm{meV}}}.$$

X_2_, representing the B exciton residing around 1.76 eV, though has a large oscillator strength compared with sidebands of exciton X, which is far detuned from exciton X and thus has negligible influence on the lower polaritons near exciton X. Here, the ${\varepsilon }_{a}=11$ and ${\varepsilon }_{c}=4$ are adopted in our simulations.

### Isofrequency surfaces

For plane waves traveling inside an anisotropic medium, the wave equation can be given by^47^

3 $$k\times \left(k\times E\right)+{k}_{0}^{2}\varepsilon E=0,$$

where *E* is the electric field, and *k* and *k*_0_ are the wavevectors in the medium and free space, respectively, and *ε* is the permittivity tensor. By applying the condition for the nontrivial plane wave solution in Eq. (3), one can obtain

4 $$\left[\begin{array}{ccc}{\varepsilon }_{{xx}}{k}_{0}^{2}-{k}_{x}^{2}-{k}_{y}^{2} & {k}_{x}{k}_{y} & {k}_{z}{k}_{x}\\ {k}_{x}{k}_{y} & {\varepsilon }_{{yy}}{k}_{0}^{2}-{k}_{z}^{2}-{k}_{x}^{2} & {k}_{y}{k}_{z}\\ {k}_{z}{k}_{x} & {k}_{y}{k}_{z} & {\varepsilon }_{{zz}}{k}_{0}^{2}-{k}_{x}^{2}-{k}_{y}^{2}\end{array}\right]=0,$$

where ${\varepsilon }_{xx}$, ${\varepsilon }_{{yy}}$, and ${\varepsilon }_{{zz}}$ are the x-, y-, and z-components of dielectric tensor, corresponding to ${\varepsilon }_{b}$, ${\varepsilon }_{a}$, ${\varepsilon }_{c}$. The allowed *k* vectors can be calculated by solving Eq. (4), giving the isofrequency surface at fixed energy.

### Numerical simulations

The angle-resolved reflectance spectra are calculated with the RCWA method based on the permittivities mentioned above. To match the dielectric environment in the experiment, the refractive index is set as *n* = 1 for the top layer (air) and *n* = 1.46 for the bottom layer (SiO_2_). For the near-field calculation, the eigenfrequency of the GMR-polariton mode at the normal direction is first confirmed by the reflection spectrum, and then the near field is calculated at the chosen frequency for this mode. For the BIC-polariton mode, because it is strictly dark at normal direction, we thus calculated the spectrum at near-normal (0.01°) and then performed the near-field calculation.

### Optical measurements

The optical measurements were performed in the home-built angle-resolved setup with a close-cycled cryostat (Montana Cryostation S50) at 4 K (see Supplementary Fig. S10). The signal was collected by an objective of NA = 0.65. For the reflectivity measurements, the differential reflectance $\frac{\Delta R}{R}$, defined by (*R*_sample_ – *R*_sub_)/*R*_sub_ where *R*_sample_ (*R*_sub_) represents the reflectance from the sample (substrate), respectively, is measured for both the pristine CrSBr flake and nanogratings. The white light was applied as the excitation field whose polarization was aligned with the b-axis of CrSBr by a linear polarizer. For the PL measurement, the laser with a wavelength of 532 nm impinged on the sample and the PL signal was collected.

## Supplementary information

Supplementary Information

Transparent Peer Review file

## Data Availability

The source data generated in this study can be accessed via the *Zenodo* repository at https://doi.org/10.5281/zenodo.21131324.

## Code availability

The codes are available from the corresponding author upon request.
