# Supplementary material for: Observation of polaritonic flat-band bound states in the continuum in a 2D magnet
Source: Nat Commun. 2026 Aug 19;17:9908. doi: 10.1038/s41467-026-76785-w (PMC13583042; doi:10.1038/s41467-026-76785-w)
Supplement: Supplementary file 1 [file 41467_2026_76785_MOESM1_ESM.pdf]

Observation of polaritonic flat-band bound states in the continuum in a 2D magnet

Fuhuan Shen[1,†], Jiahao Ren[1,†], Zhiyi Yuan[2,3], Kai Wu[1], Sai Yan[1], Kunal Prasad[1], Hai Son Nguyen[2,4], Rui Su[1,3*]

1. Division of Physics and Applied Physics, School of Physical and Mathematical Sciences, Nanyang Technological University, Singapore 637371, Singapore.
2. CNRS-International-NTU-Thales Research Alliance (CINTRA), IRL 3288, Singapore 637553
3. School of Electrical and Electronic Engineering, Nanyang Technological University, Singapore, Singapore.
4. Ecole Centrale de Lyon, INSA Lyon, Université Claude Bernard Lyon 1, CPE Lyon, CNRS, INL, UMR5270, Ecully 69130, France

† These authors contribute equally

* Corresponding author. Email: surui@ntu.edu.sg

Contents

A. Generation of GMR and BIC modes in the periodic grating structure

B. Theoretical model at strong (SC) and ultra-strong (USC) coupling regime

C. Influence of geometric parameters on GMR- and BIC-bands

D. Measured results of the CrSBr grating with *ff*=0.5

E. Measured results of 14 nm CrSBr samples

F. Model fits for high-order upper and lower polaritons in CrSBr gratings

G. Angular range of the flat bands for second-order polaritonic BIC-bands

H. Optical path setup for angle-resolved measurements

I. Measured spectra for high-order modes and HEPs at some fixed k_x values.

J. TMM fitting for the reflectance spectra of a CrSBr flake along a- and b-axes.

K. Theory and numerical mapping of folded HEPs in patterned CrSBr

A. Generation of GMR and BIC modes in the periodic grating structure

A theoretical model was developed in previous work to describe the generation of GMR and BIC modes in the periodic grating structures, which can be illustrated in two steps (Fig. **S1**). Though the propagating guided modes lie below the light cone of the vacuum which thus cannot be coupled to the radiative continuum, the periodic structure would out-couple these counter-propagating guided modes to the free-space due to the modified momentum $\beta^{\pm} = k_x \pm m\frac{2\pi}{a}$, resulting in the photonic band folding. Here, m represents the diffraction order and k_x is the in-plane momentum. The dispersion can be thus given by

$$E_m^{\pm} = E_m \pm v_g k_x \,, \qquad \text{(S1.)}$$

where E_m represents the energy at Γ point and v_g is the group velocity. The dispersions $E_m^{\pm}$ are indicated by the red and blue lines in the energy band (Fig. **S1a**). The interactions between these two counter-propagating guide modes arise from the Bragg scattering and their coupling with the radiative continuum, which can be described by a non-Hermitian Hamiltonian[1,2], i.e.,

$$H^m = \begin{bmatrix} E_m^+ & \xi \\ \xi & E_m^- \end{bmatrix} + i\gamma_r \begin{bmatrix} 1 & 1 \\ 1 & 1 \end{bmatrix}. \qquad \text{(S2.)}$$

The first term of H^m represents the energy of uncoupled m-order propagating mode and their coupling through diffraction (ξ represents the corresponding coupling strength). The second term describes the radiative loss γ_r and the coupling via the radiative continuum. The eigenvalues of the H^m can be solved, which will give the eigen-energies due to the coupling:

$$U_{\pm} = E_m + i\gamma_r \pm \sqrt{(v_g k_x)^2 + (\xi - i\gamma_r)^2}. \qquad \text{(S3.)}$$

At k_x=0 (Γ point), these two eigen-energies can be calculated as $U_+ = E_m + \xi$ and $U_- = E_m - \xi + 2i\gamma_r$, respectively, indicating an open energy gap due to the coupling (Fig. **S1b**). Moreover, their radiation losses are given by the imaginary parts of eigen-energies. Notably, the sum of $\gamma_{\pm}$ is conserved: $\gamma_+ + \gamma_- = \mathrm{Im}[U_+] + \mathrm{Im}[U_-] = \mathrm{Im}[U_+ + U_-] = 2\gamma_r$. For BIC modes, $\gamma_+ = 0$ at $k_x = 0$ μ m^{-1}, corresponding to an infinite Q factor of true BICs at the Γ point. As k_x increases, BICs evolve into quasi-BICs, with corresponding increases in γ_+ due to enhanced asymmetry, which consequently leads to a decrease in the Q factors. Conversely, for the GMR mode, $\gamma_- = 2\gamma_r$ reaches its maximum at $k_x = 0$ and gradually decreases as k_x increases, leading to the opposite trend of Q factors for the GMR mode compared to the BIC mode.

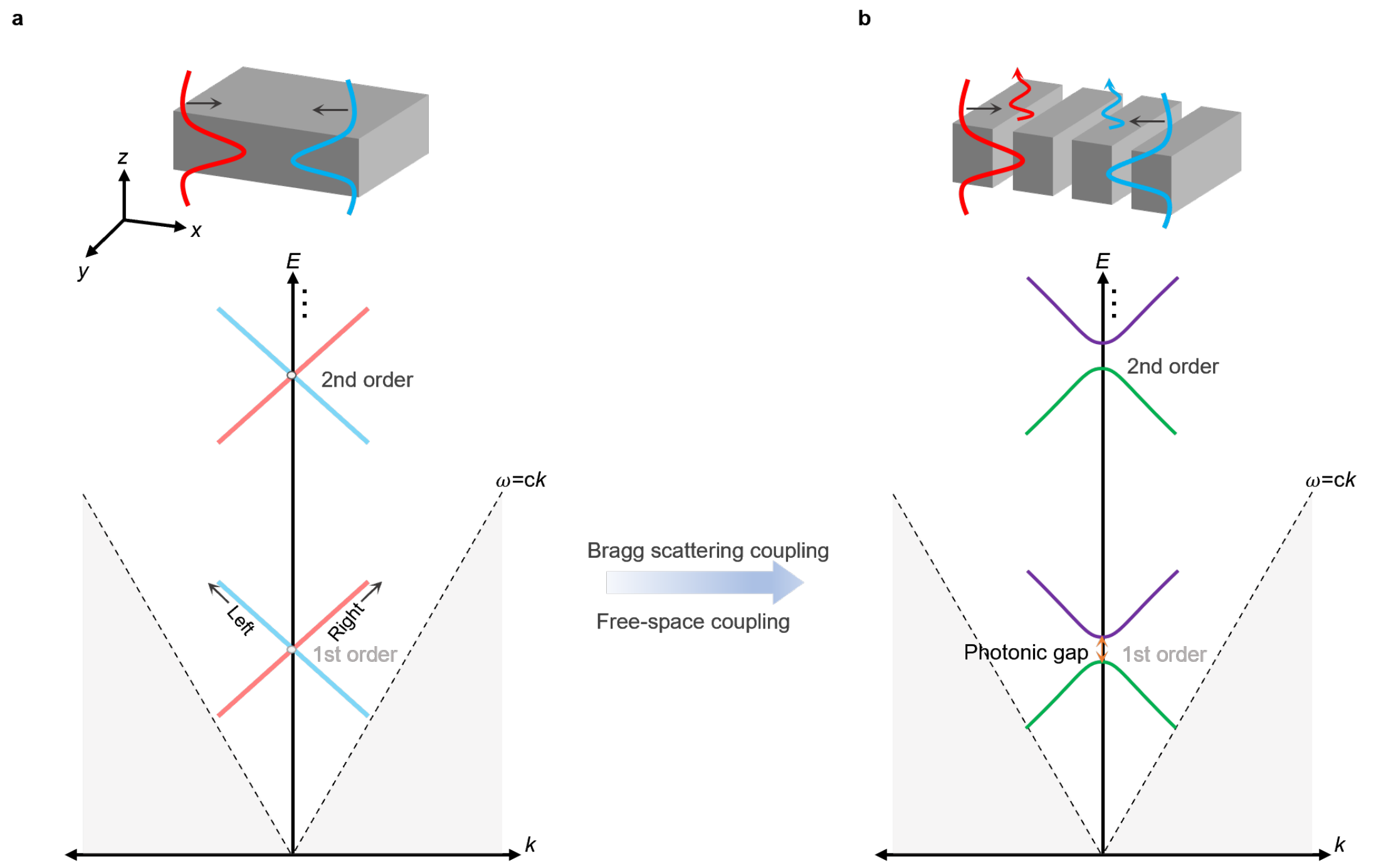

Fig. S1 | Schematic illustration of the generation of GMR and BIC modes due to the diffractive coupling. (**a**) Schematic illustration of uncoupled first and second-order counter-propagating modes above the light line. (**b**) Corresponding BIC and GMR modes due to the Bragg scattering in the periodic gratings.

B. Theoretical model at strong (SC) and ultra-strong (USC) coupling regime

B.1 SC and USC Hamiltonian and eigenvalues

The Hamiltonian describe the light-matter interaction can be composed of three terms, i.e.,

$$H = H_{pho} + H_{exc} + H_{int}. \tag{S4.}$$

Here, $H_{pho} = \omega_c(\frac{1}{2} + \hat{a}^\dagger\hat{a})$ and $H_{exc} = \omega_a(\frac{1}{2} + \sigma_z)$ represent the uncoupled photonic and exciton modes, respectively, and $H_{int} = g(\hat{a}^\dagger + \sigma_+) \cdot h.c.$ represents the interaction term. $\hat{a}^\dagger$ ($\hat{a}$, its Hermitian counterpart) is the increasing (annihilation) operator for photons and σ_+ (σ_-, its Hermitian counterpart) is the increasing (decreasing) operator for excitonic transition. ω_c (ω_a) and g represent the uncoupled cavity (exciton) resonance and their mutual coupling strength, respectively. The natural units are adopted and *h.c.* represents the Hermitian conjugation for short. Typically, for the strong coupling regime, the rotation wave approximation (RWA) is adopted, where the fast rotation term is abandoned, resulting in the simplified interaction term $H_{int} = g(\hat{a}\sigma_+ + h.c.)$. Using the basis $|0, e\rangle$ and $|1, g\rangle$ where the first index indicates the photonic number and second index refers to the excited (e) or ground (g) state of exciton, one can obtain the matrix form of Hamiltonian as

$$H=\begin{bmatrix}\omega_c & g\\ g & \omega_a\end{bmatrix}+i\begin{bmatrix}\gamma_c & 0\\ 0 & \gamma_a\end{bmatrix}, \tag{S5.}$$

where γ_c (γ_a) represents the decay rate of the cavity (exciton). The eigen-energies at zero detuning can thus be calculated as

$$\omega_{\pm}=\omega_c+i\frac{\gamma_c+\gamma_a}{2}\pm\sqrt{g^2-\frac{(\gamma_c-\gamma_a)^2}{4}}. \tag{S6.}$$

The system is at the strong coupling regime when the term $(g^2-\frac{(\gamma_c-\gamma_a)^2}{4})>0$, leading to the mode splitting and generation of lower and upper polaritons (LPs and UPs). Correspondingly, the evolution of polaritonic dispersion can be given by

$$\omega_{\pm}=\frac{\omega_c+\omega_a}{2}\pm\sqrt{g^2-\frac{\Delta^2}{4}}, \tag{S7.}$$

where the detuning is defined as $\Delta=(\omega_c+i\gamma_c-\omega_a-i\gamma_a)$.

However, when the system is at the ultra-strong (USC) coupling regime, i.e., $\frac{g}{\omega_a}>0.1$, the fast-rotation term and so-called A^2 term arise, resulting in the modified interaction Hamiltonian[3,4]

$$H_{int}=g(\hat{a}\sigma_{+}+\hat{a}^{\dagger}\sigma_{+}+h.c.)+\frac{g^2}{\omega_a}(\hat{a}^{\dagger}+\hat{a})^2. \tag{S8.}$$

The eigenvalues $\omega_{\pm}$ for above Hamiltonian (eq. (S8)) can be given by the positive solutions of the bi-quadratic equation, i.e.,

$$({\omega_{\pm}}^2-{\omega_c}^2)({\omega_{\pm}}^2-{\omega_a}^2)-\frac{4g^2{\omega_{\pm}}^2\omega_c}{\omega_a}=0, \tag{S9.}$$

which can be numerically calculated.

In both above models, we consider GMR or BIC independently coupled with excitons and adopt g_1 (GMR-polaritons) and g_2 (BIC-polaritons) to represent their respective coupling strengths.

B.2 Hopfield coefficients

The Hopfield coefficients of cavity photon (α) and exciton (β) for upper or lower polariton, which satisfy $|\alpha|^2+|\beta|^2=1$, can be calculated as

$$|\alpha_{\pm}|^2=\frac{1}{2}(1\pm\frac{\Delta}{\sqrt{\Delta^2+4g^2}}), \tag{S10.}$$

and

$$|\beta_{\pm}|^2 = \frac{1}{2}(1 \mp \frac{\Delta}{\sqrt{\Delta^2 + 4g^2}}). \qquad (S11.)$$

B.3 Comparison of fittings by USC and SC models

Figure **S2** compares the fitting results from two models using the numerical data presented in Fig. **1e** of the main text. The USC model (Fig. **S2a**) demonstrates a good alignment with the numerical results. In contrast, the SC model, utilizing the same parameters as USC model, shows a clear deviation (Fig. **S2b**). Although optimizing the parameters (Fig. **S2c**) improves the alignment between the SC model and the numerical results, notable deviations remain, particularly for BIC-polaritons.

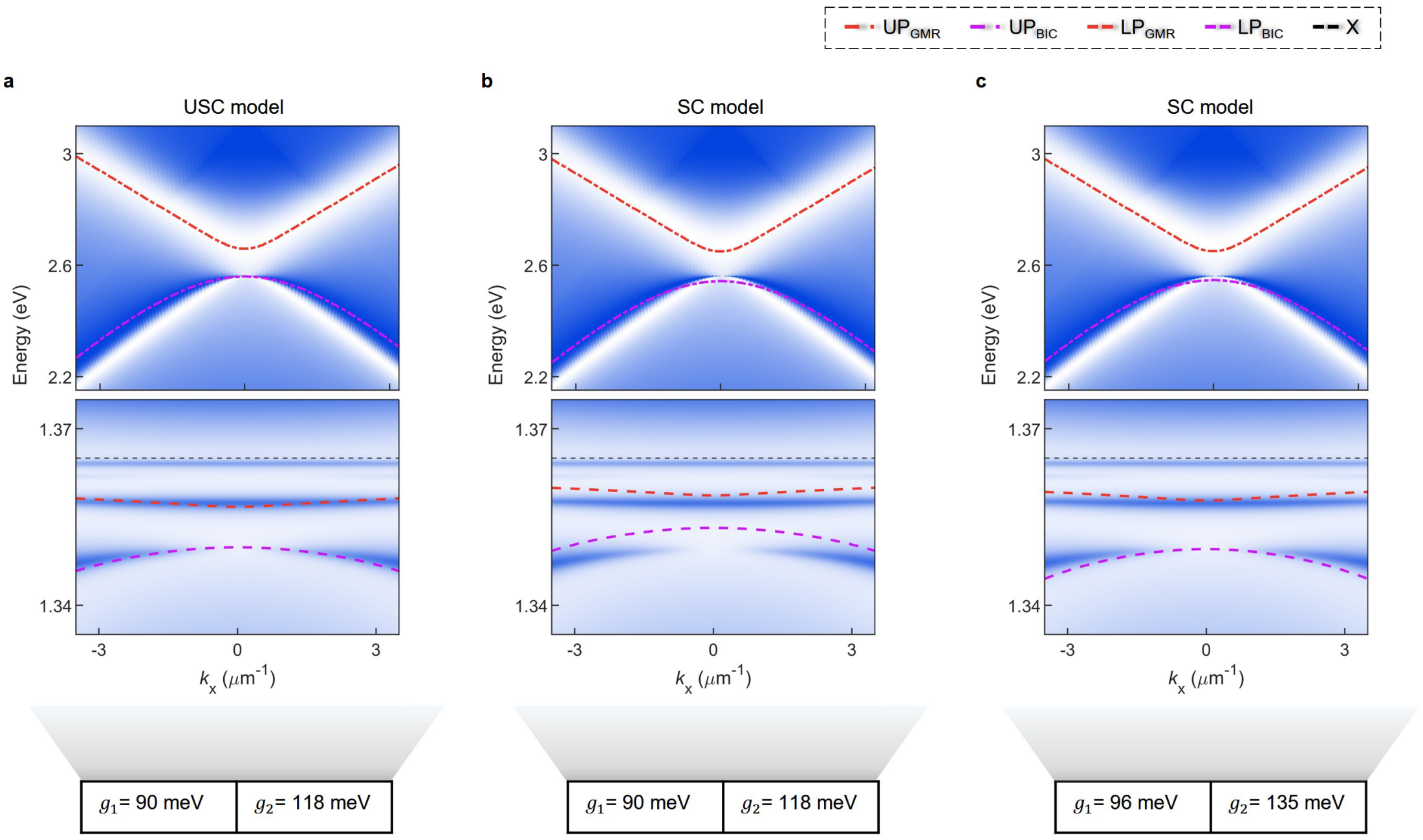

Fig. S2 | Comparison of fittings by USC and SC models. (**a**) Fitting by USC model. The bottom panel shows the corresponding coupling strengths: g_1 represents the coupling strength between excitons and GMR modes; g_2 represents coupling strength between excitons and BIC modes. (**b-c**) Fittings by SC model using the same (**b**) parameters as (**a**) and optimized parameters (**c**).

Based on the USC model, we extracted the corresponding coupling strengths from the measured result (Figure **2h** in the main text), as shown in Figure **S3**. The extracted values ($g_1 \approx 85$ meV and $g_2 \approx 115$ meV) align with those from the simulation results. Because of the large detuning (> 1 eV), by substituting the coupling strength in Eq. (10) and (11), the corresponding Hopfield coefficients can be calculated for UP_{BIC} (upper BIC-polaritons) as $|\alpha_+| > 0.95$, and for LP_{BIC}, $|\beta_-| > 0.95$.

Additionally, Table **S1** presents a comparison of coupling strengths between our work and other cavity systems utilizing CrSBr. The coupling strength $g = 115$ meV observed in our study is comparable to that of Fabry-Pérot (FP) cavity systems where CrSBr overlaps with the cavity modes, and exceeds that found in CrSBr-photonic crystal (PC) hybrid systems employing an external PC. The variations in coupling strength between our work and others can be attributed to several factors: (i) differences in the

thickness of the CrSBr samples, (ii) varying degrees of field confinement due to different optical modes, and (iii) the extent of overlap between CrSBr and the optical modes.

Despite the large difference in layer thickness, the coupling strength associated with our ~25-nm BIC structure is close to that of the ~580-nm (Ref. 5) or ~462-nm (Ref. 6) FP cavity. To provide quantitative analysis of the coupling strength, we first calculated the mode distributions (without excitonic Lorentz oscillator, as a simulation using full resonant dielectric function already captures the hybrid exciton–polariton modes) separately for the 25 nm–BIC structure (Fig. **S4a-b**) and the 580 nm–FP cavity (Fig. **S4c-d**), and then investigated how the coupling strengths depend on these near-field distributions. The coupling strength can be calculated as $g \propto \mu \cdot E \propto \sqrt{N/V}$. Here, N is the exciton number, which is proportional to the geometric volume of structure. For instance, for the grating structure (one unit) in Fig. **S4a**, N is proportional to the product of width w, layer thickness t, and length l (which is infinite along the y-axis but can be cancelled out by V in the expression $\sqrt{N/V}$). V is the effective mode volume, which can be evaluated as $V = \frac{\int \varepsilon(\mathbf{r})|E(\mathbf{r})|^2\, d^3\mathbf{r}}{\max[\varepsilon(\mathbf{r})|E(\mathbf{r})|^2]}$. Based on their respective near-field distributions, we can estimate the relative coupling strengths as $g_{\text{BIC}} \propto \sqrt{\frac{N_{\text{BIC}}}{V_{\text{BIC}}}} \approx 0.8405, g_{\text{FP}} \propto \sqrt{\frac{N_{\text{FP}}}{V_{\text{FP}}}} \approx 1.31$, yielding $g_{\text{FP}}/g_{\text{BIC}} \approx 1.56$.

Although reducing (increasing) the thickness decreases (increases) the exciton number contributing to the coupling (i.e., N), the effective mode volume V changes in the same direction. As a result, the decrease (increase) in mode volume partially compensates the thickness-dependent change in N, so that the net coupling strength remains comparable. The slight mismatch between the measured ratio ($\frac{g_{\text{FP}}}{g_{\text{BIC}}} = \frac{120}{115} \sim 1.04$) and the mode-volume estimation can be attributed to small differences in the fitted dielectric parameters used in our simulations and in Ref. 5.

It is worth mentioning that our estimated ratio of 1.56 is consistent with coupling strength reported in Ref. 6 where $g_{\text{FP}} \sim 169\,\text{meV}$ gives rise to a ratio of $\frac{169}{115} \approx 1.47$, suggesting our extracted coupling strengths are reasonable.

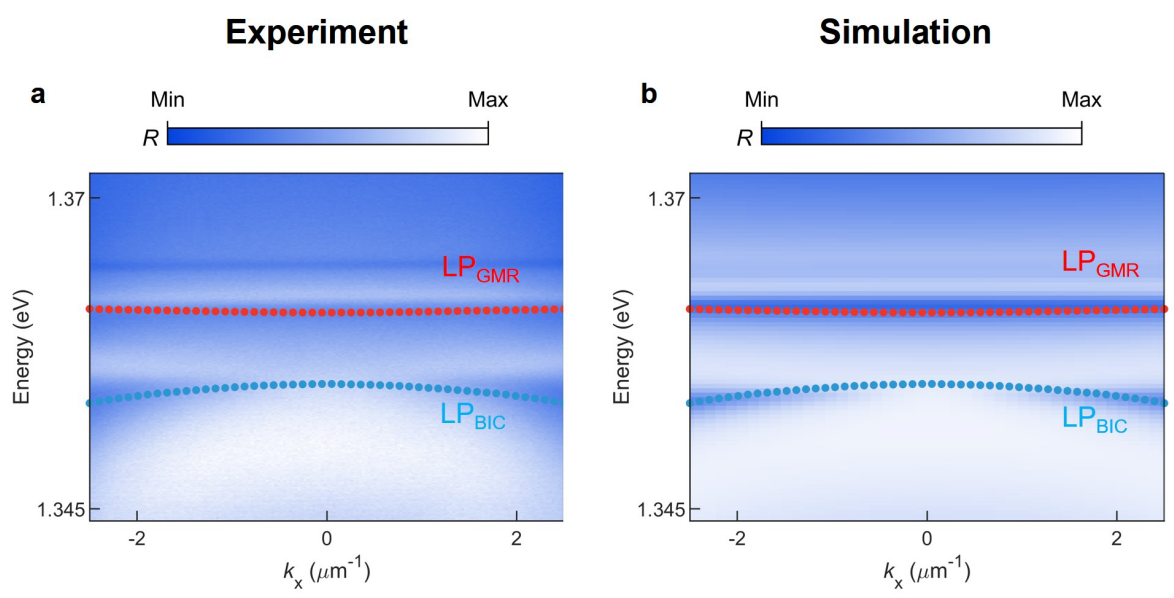

Fig. S3 | Mode fittings with the measured results. (**a**) Measured angle-resolved reflectance spectra

(data from **Fig. 2d** in the main text). The red and blue dots indicate the fitted dispersions of the LP_{GMR} and LP_{BIC} branches, respectively. The extracted coupling strengths are $g_1 = 85$ meV and $g_2 = 115$ meV, respectively. (**b**) Simulated results corresponding to (**a**).

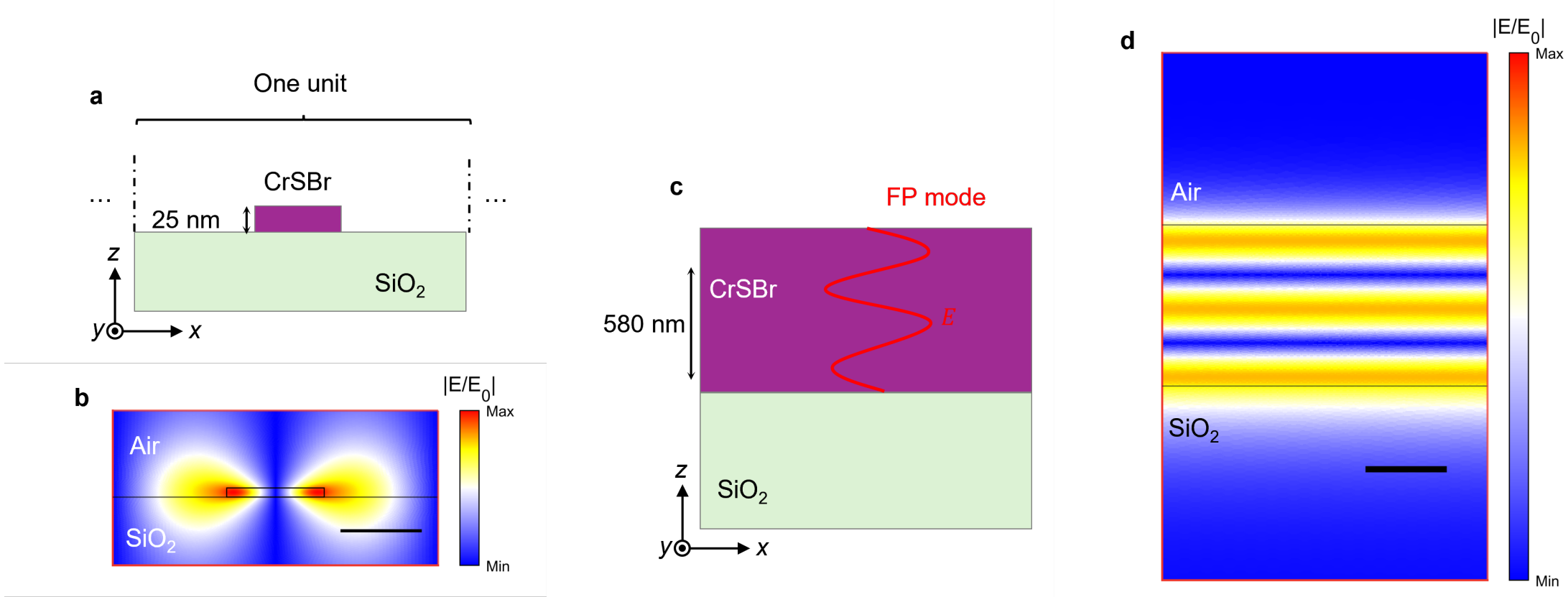

Fig. S4 | Mode distributions of the BIC and FP modes. (**a**) Schematic of the 25-nm CrSBr grating supporting a BIC mode. (**b**) Near-field distribution of the photonic BIC mode with the excitonic Lorentz oscillator removed. (**c**) Schematic of the 580-nm CrSBr flake supporting FP modes. (**d**) Near-field distribution of one photonic FP mode with the excitonic Lorentz oscillator removed.

References	Cavity types	Thickness	Coupling strength
Ref. 5	Self-hybridized Fabry-Pérot cavity	~ 580 nm	~120 meV
Ref. 6	Fabry-Pérot cavity	~ 462 nm	~169 meV
Ref. 7	GMR (external Si_3N_4 PC)	7 layers (~ 6 nm)	~ 58 meV
Our work	Self-hybridized BICs	~ 26 nm	~115 meV

Table S1 | Comparison of coupling strengths between our work and previous work.

C. Influence of geometric parameters on GMR- and BIC-bands

Akin to traditional PCs, geometric parameters such as CrSBr thickness and grating period significantly

influence the optical behavior of GMR and BIC modes. Increasing the CrSBr thickness leads to a clear red shift of both GMR- and BIC-bands, as shown in Figure **S5a**. Moreover, the energy gap between these two modes widens with increasing thickness. Moreover, a second-order mode emerges when the thickness surpasses 20 nm.

Regarding the period, both BIC-band and GMR-band exhibit a noticeable red shift as the period increases (Fig. **S5b**), while the BIC-band showing a more pronounced shift compared to the GMR-band. When the period exceeds 400 nm, first-order BIC-band becomes significantly angularly dispersive. In addition, the higher-order branches emerge at larger period. The impact of the filling factor is illustrated in Figure **S5c**, which is discussed in detail in the main text.

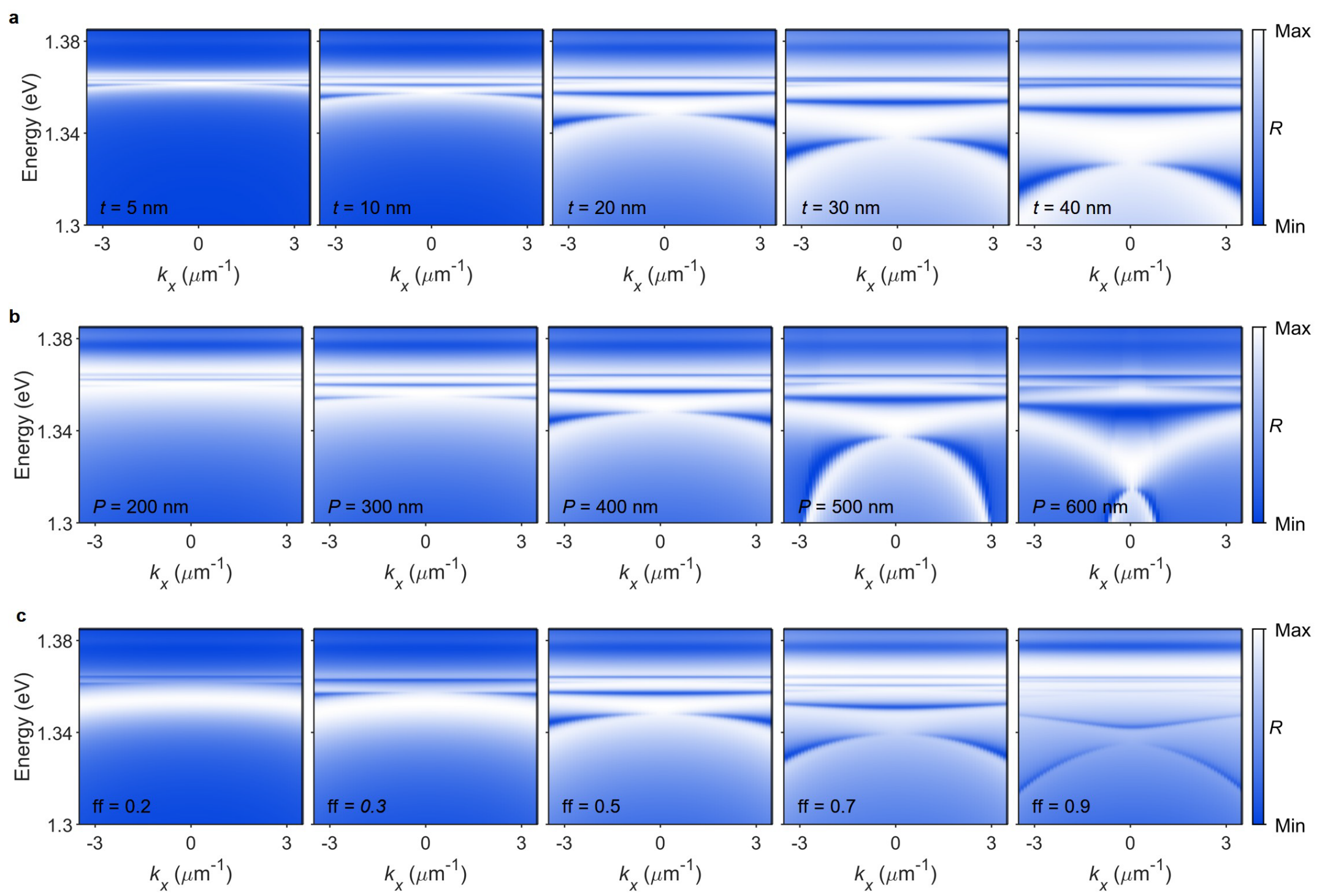

Fig. S5 | Evolution of polaritonic GMR- and BIC-bands with geometric parameters. (**a**) Dependence on the CrSBr thickness *t*, where *P* = 400 nm and *ff* = 0.5. (**b**) Dependence on the period *P* where *t* = 20 nm and ff = 0.5. (**c**) Dependence on the filling factor *ff*, where *P* = 400 nm and *t* = 20 nm.

D. Measured results of the CrSBr grating with *ff*=0.5

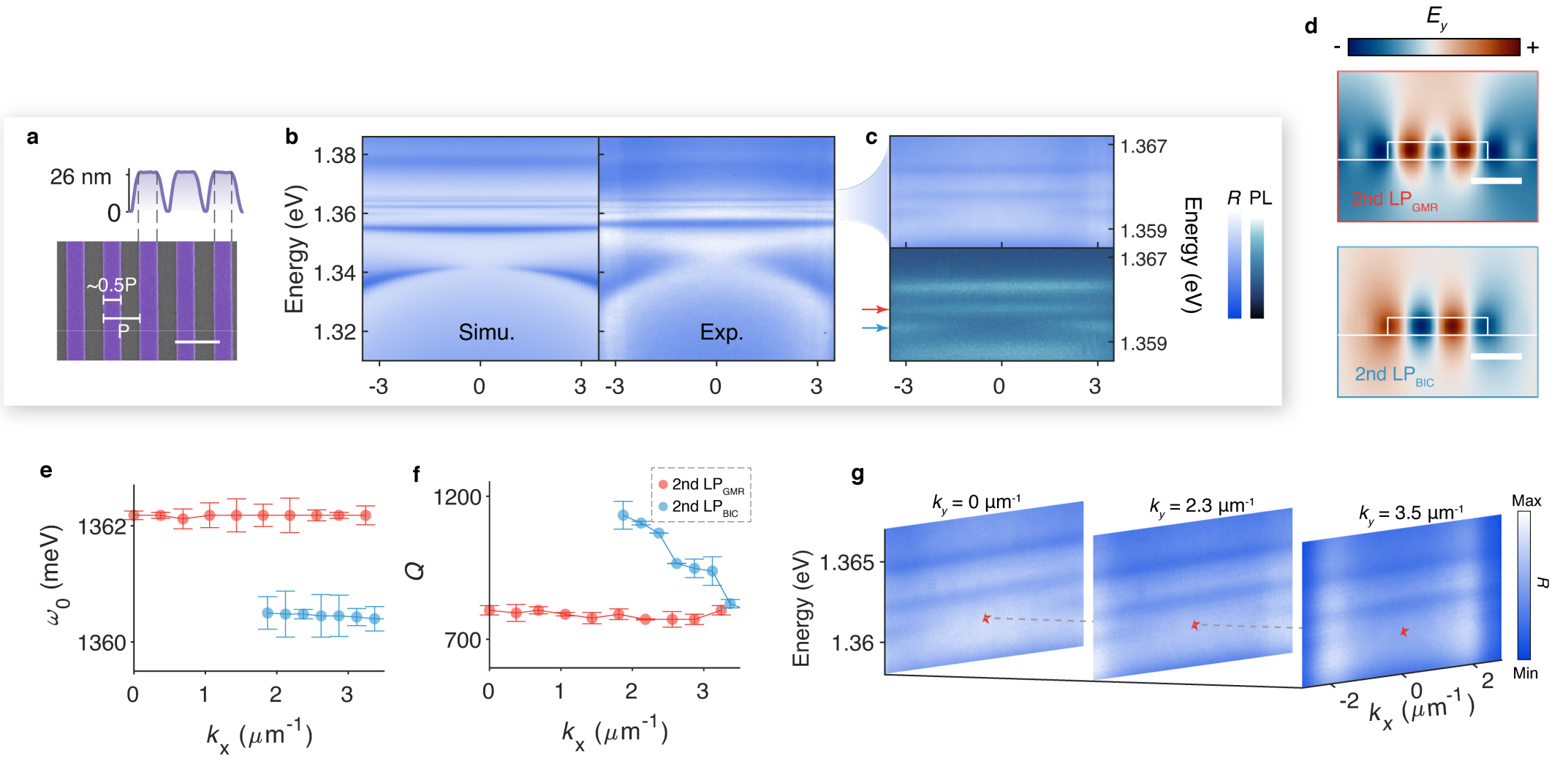

Fig. S6 | Second-order LP$_{BIC}$ and LP$_{GMR}$ bands for the CrSBr grating with a filling factor of 0.5. (**a**) SEM image of CrSBr grating showing a filling factor of 0.5 and its corresponding AFM profile. (**b**) Corresponding simulated (left) and measured (right) angle-resolved reflectance spectra for CrSBr gratins in (**a**). (**c**) Zoom-in reflectance spectra and corresponding PL spectra, showing second-order LP$_{BIC}$ (noted by a blue arrow) and LP$_{GMR}$ (noted by a red arrow). (**d**) Calculated near field distributions for second-order LP$_{BIC}$ (bottom) and LP$_{GMR}$ (top), respectively. (**e-f**) Extracted resonant frequencies (**e**) and corresponding Q factors (**f**) for the PL data in (**e**). (**g**) Angle-resolved reflectance spectra at k_y=0, 2.3, and 3.5 μm^{-1}, respectively.

E. Measured results of a 14-nm-thickness CrSBr sample

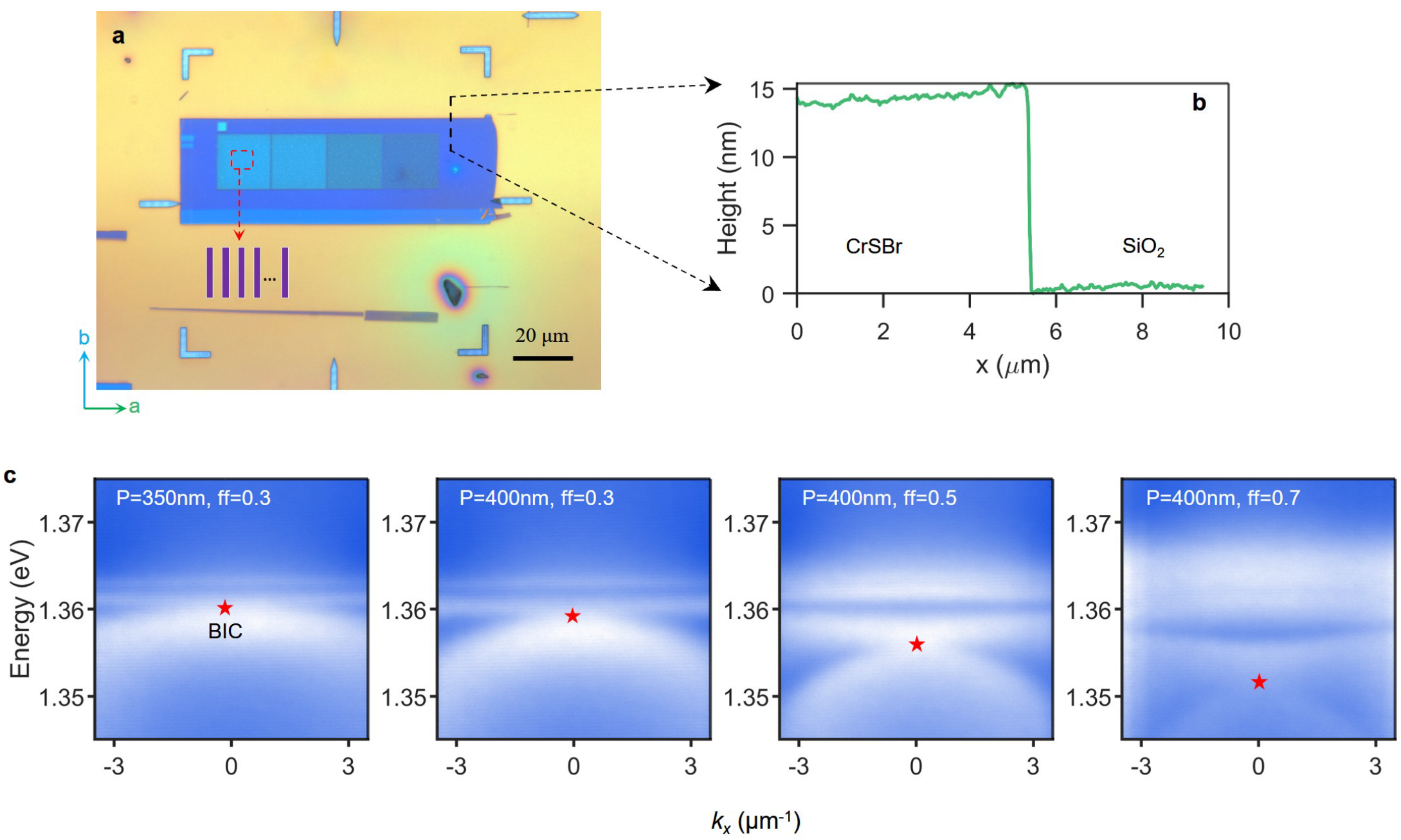

Fig. S7 | TE polaritonic bands measured from CrSBr gratings of 14 nm thickness. (**a**) Microscopic image of the 14 nm CrSBr sample. (**b**) AFM profile of CrSBr flake showing a thickness of around 14 nm. (**c**) Measured angle-resolved reflectance spectra from CrSBr gratings with the left-to-right sequence corresponding to the gratings in (**a**). Red stars indicate the position of BIC-polaritons.

In addition to the sample shown in Figure **2b** of the main text, we fabricated and measured other CrSBr grating samples with varying geometric parameters. As illustrated in Figure **S7**, the thickness of the CrSBr layer is approximately 14 nm (Fig. **S7a-b**). The measured reflectance spectra also exhibit polaritonic BIC-band; however, the optical contrast is diminished (Fig. **S7c**). While the first-order mode can be clearly distinguished, the higher-order modes appear relatively blurred.

F. Model fits for high-order upper and lower polaritons in CrSBr gratings

Figure **S8** presents the calculated reflectance spectra for both the first- and second-order upper and lower polaritonic bands. Compared to the first-order photonic BIC- and GMR-bands, the second-order ones lie at much higher energy, rendering the corresponding LP bands being flattened over a wider angular range.

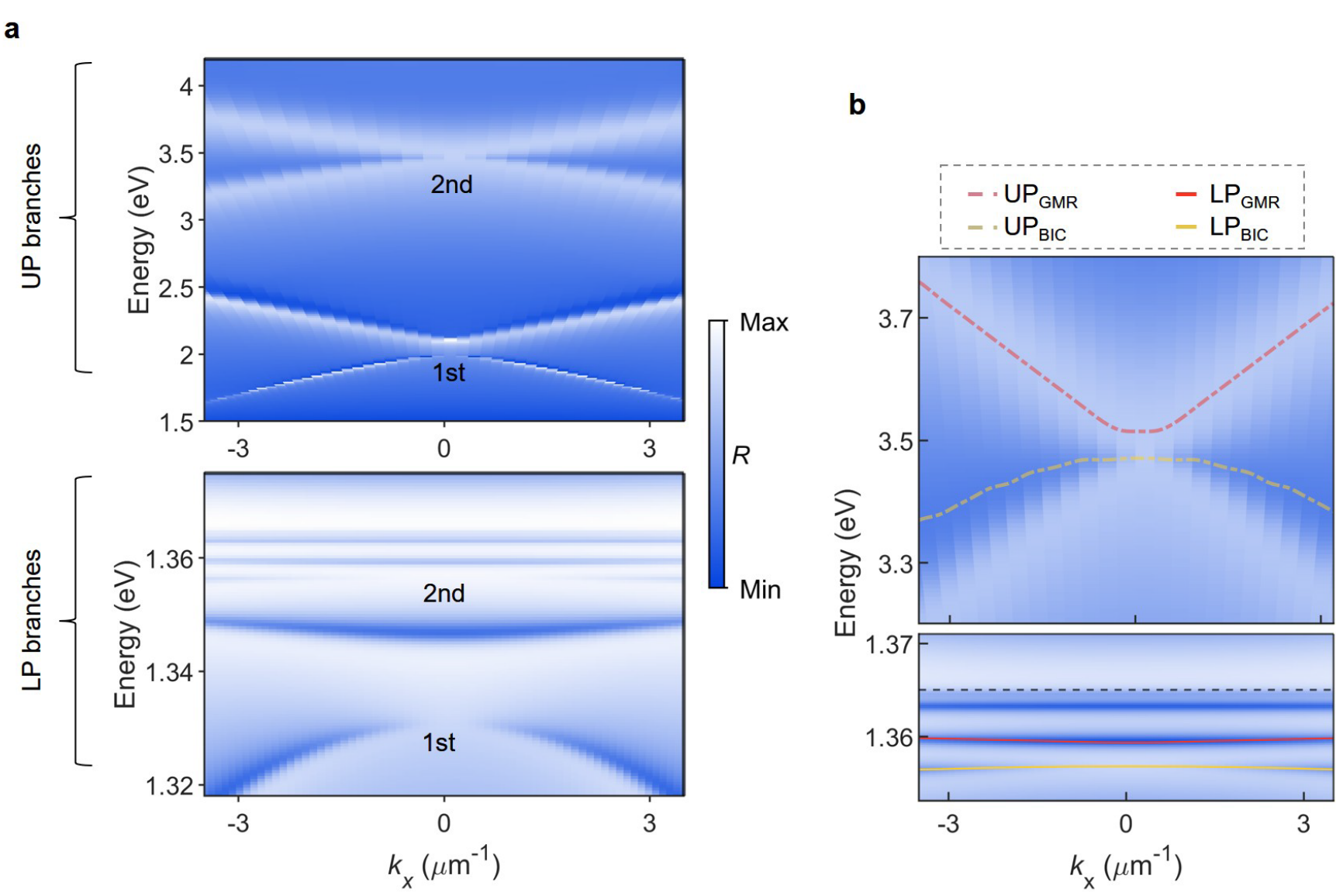

Fig. S8 | High-order upper and lower polaritonic bands. (**a**) Upper panel: dispersions for first- and second-order upper polaritonic bands. Lower panel: dispersions of the first- and second-order lower polaritonic bands. (**b**) Polaritonic dispersions fitted by the USC model. The coupling strength for second-order exciton-BIC polaritons is extracted as 110 meV. Geometric parameters: P=400 nm, t = 25 nm, ff = 0.7.

G. Angular range of the flat bands for second-order polaritonic BIC-bands

The maximum angle of flat-band polaritons, as one of the key parameters for the performance of flat-band modes, is limited by collection angle in the experiment using an objective with numerical aperture NA=0.65. However, the numerical calculation by the RCWA indicates (Fig. **S8**) the maximum angle of

flat-band polariton is merely limited by the light line, which shows fundamentally different and remarkably better performance compared to the previous systems, such as those based on the broken in-plane systems[8], or twisted alignment[9].

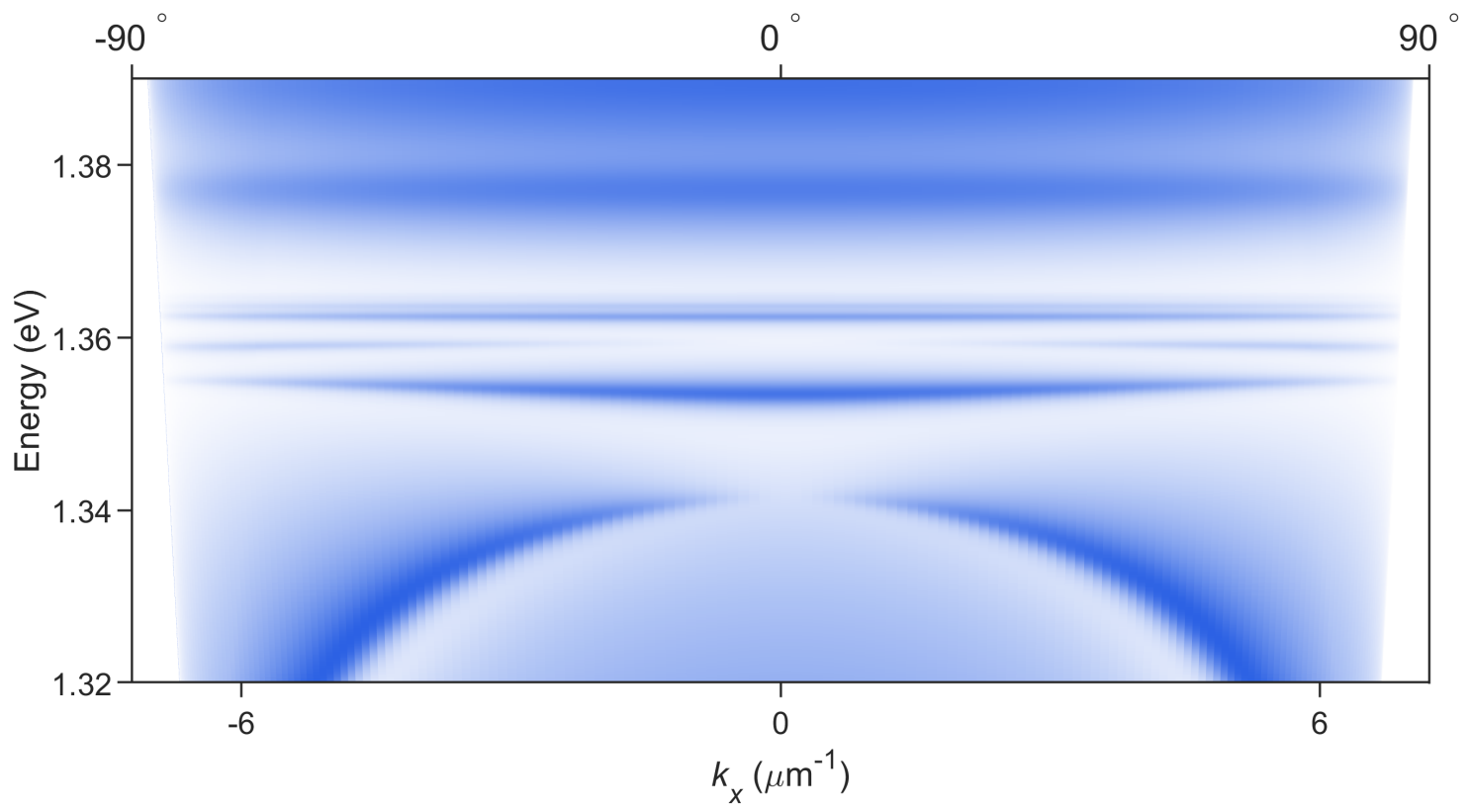

Fig. S9 | Performance of second-order polaritonic flat band in momentum space. Calculated angle-resolved reflectance CrSBr grating of ff=0.5 with angle extended from 0 to 90 degrees.

H. Optical path setup for angle-resolved measurements

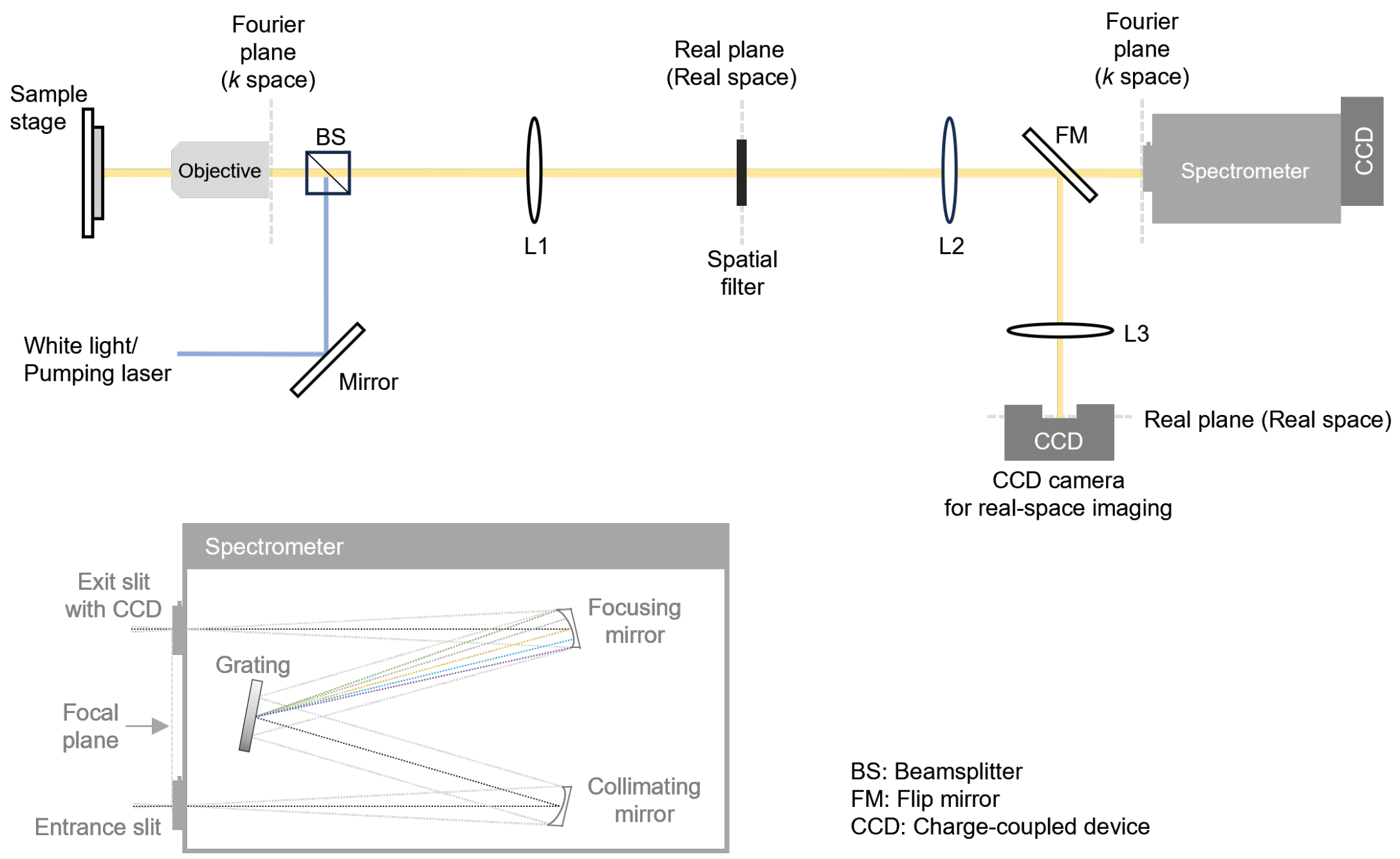

Fig. S10 | Schematic optical path for the angle-resolved measurements. In the reflectance measurements, white light is first directed through a mirror and a beam splitter (BS) before being focused on the sample by the objective lens. The reflected signal is then collected by the same objective and passes sequentially through the BS, lens L1, and lens L2, before ultimately reaching the spectrometer. A flip mirror is incorporated to allow for checking the real-space image, facilitating adjustments during the measurements. The entrance slit is used to select specific values of k_y.

I. Measured spectra for high-order modes and HEPs at some fixed k_x values.

To better compare different behaviors of polaritonic BIC- and GMR-bands with respect to the in-plane momentum, in addition to using the pseudo-color map showcased in the main text, we also present the spectra at some specific k_x values, as shown in Figure **S10**.

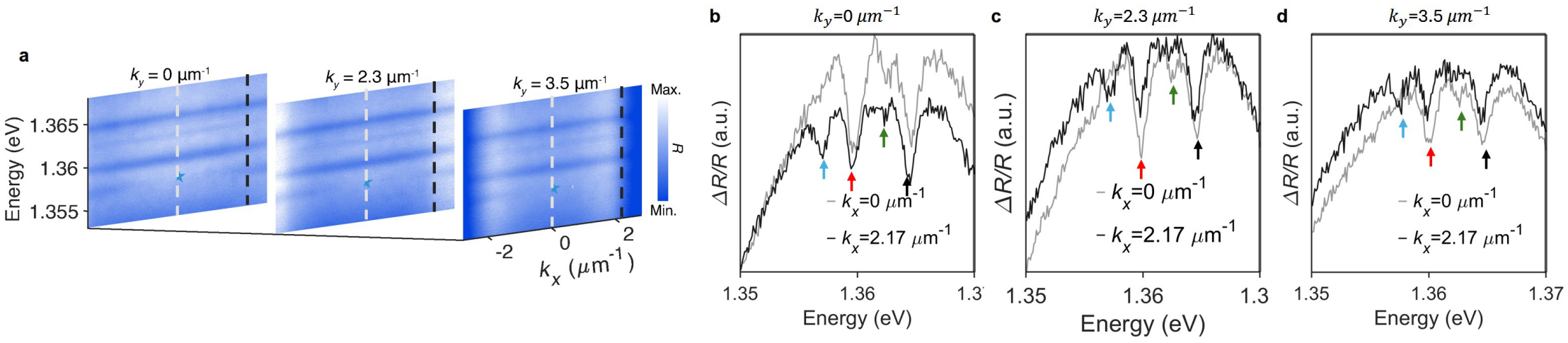

Fig. S11 | (**a**) Angle-resolved reflectance spectra for high-order GMR- and BIC-polaritons at various k_y values. Gray and black dashed lines indicate the positions at $k_x = 0$ and 1.8 μm^{-1}, respectively. (**b-d**) Extracted spectra at fixed k_x=0 and 2.17 μm^{-1}, corresponding to the data presented in (**a**).

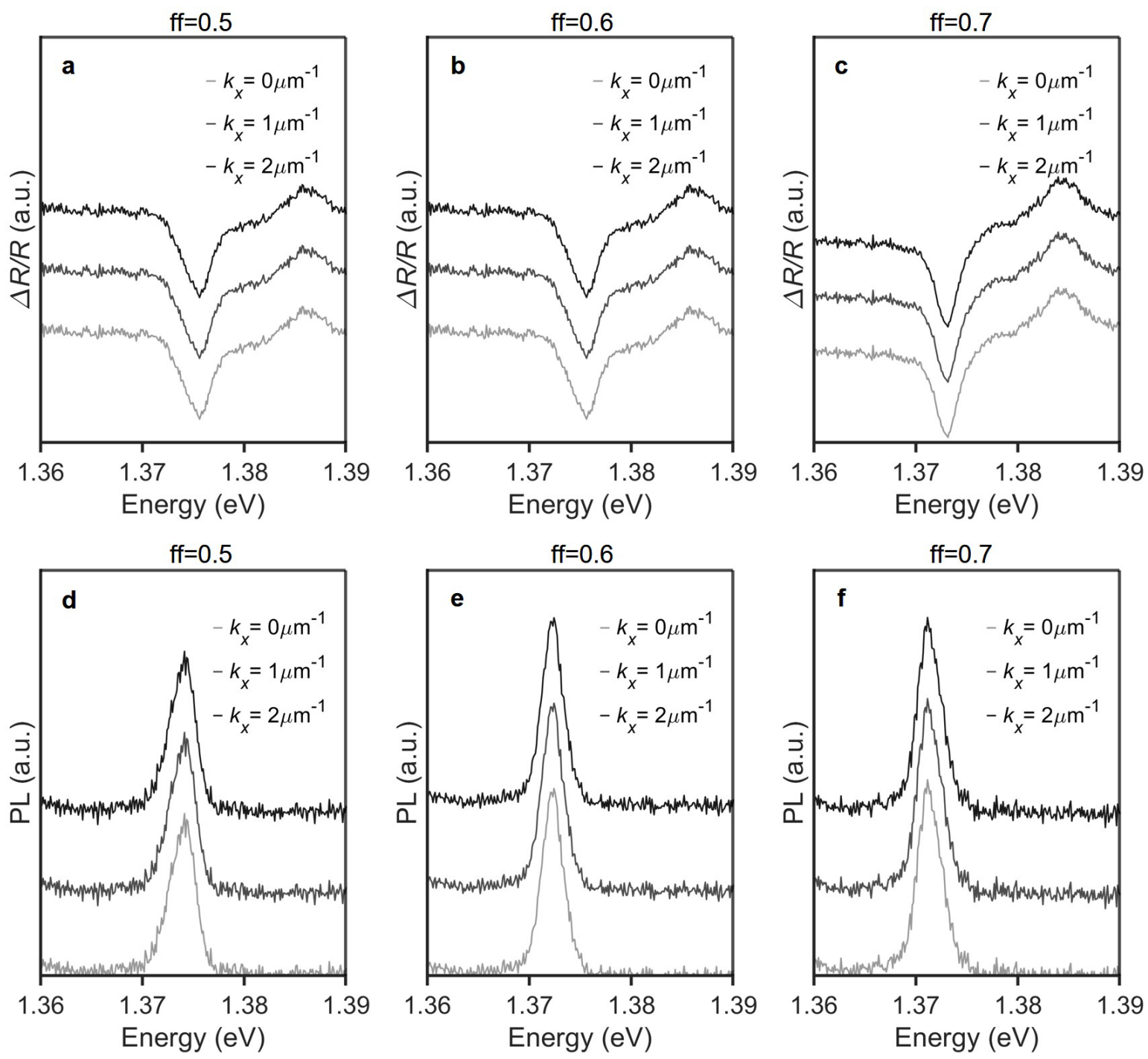

Fig. S12 | (**a-c**) Reflectance spectra of HEP modes at k_x =0, 1, and 2 μm^{-1}, respectively, for filling factor of 0.5 (**a**), 0.6 (**b**), and 0.7 (**c**), respectively. (**d-f**) PL spectra of HEP modes at k_x =0, 1, and 2 μm^{-1}, respectively, for filling factor of 0.5 (**d**), 0.6 (**e**), and 0.7 (**f**), respectively. Above data are extracted from Figure **4e** and **4f** in the main text.

J. TMM fitting for the reflectance spectra of a CrSBr flake along a- and b-axes.

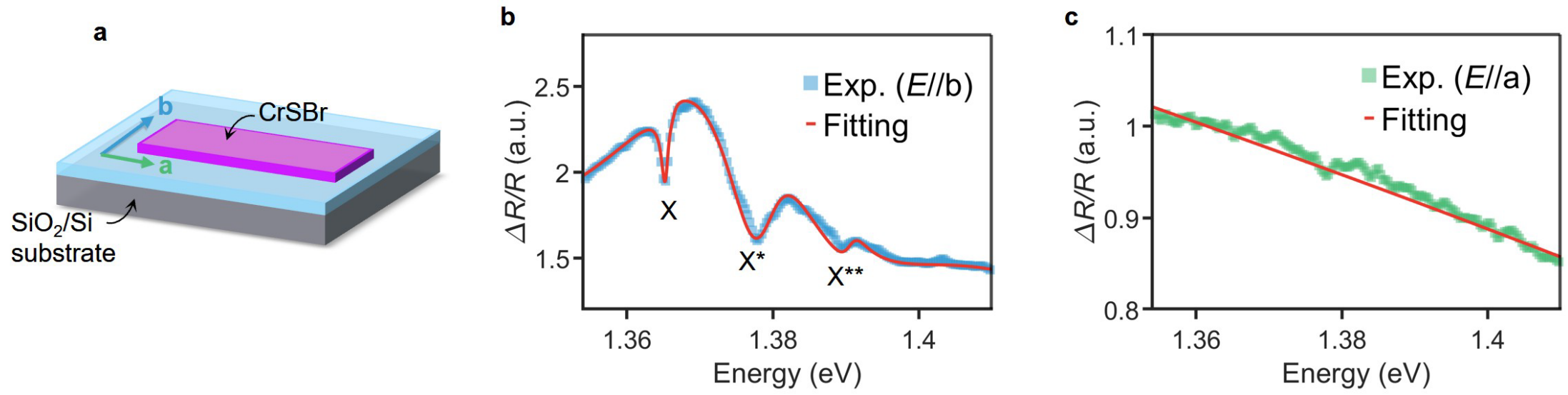

Fig. S13 | Comparison of the reflectance spectra and fittings in a CrSBr flake. (**a**) Schematic setup of CrSBr flake on the SiO_2 (300 nm)/Si substrate. (**b**) Comparison of the measured reflectance spectra (blue squares) along the *b*-axis and the fitting (red line) by transfer matrix method. (**c**) Same as (**b**) but along the a-axis.

K. Theory and numerical mapping of folded HEPs in patterned CrSBr

K.1 Anisotropic dielectric tensor of CrSBr

As indicated in the Method part in main text, we model CrSBr by a diagonal dielectric tensor $\boldsymbol{\varepsilon}(\omega)$ = diag($\varepsilon_b(\omega)$, ε_a, ε_c), with $\varepsilon_a = 11$ and $\varepsilon_c = 4$. The strongly dispersive component is the *b*-axis permittivity, described by a multi-Lorentz excitonic model. The hyperbolic exciton-polariton (HEP) regime appears when the in-plane component becomes negative while the out-of-plane com- ponent remains positive:

$$\mathrm{Re}[\varepsilon_b(\omega)] < 0, \varepsilon_c > 0. \qquad (S12.)$$

This mixed-sign tensor is the basic condition for hyperbolic TM polaritons in the (*x, z*) plane. This description is consistent with the dielectric picture used in the previous work on HEPs with CrSBr[10].

K.2 TM nature of the HEP

For propagation direction along *x*-axis, the HEP exists only in the TM sector. The reason is that the hyperbolic response is associated with the sign change of the dielectric tensor in the (*x, z*) sector: $\varepsilon_x = \varepsilon_b(\omega) < 0$ and $\varepsilon_z = \varepsilon_c > 0$. A TM wave probes both of these tensor components because its electric field has components in the (*x, z*) plane. It can therefore experience the hyperbolic isofrequency surface and support a HEP.

Additionally, for very thin CrSBr slabs (< 30 nm), the relatively small out-of-plane dielectric response makes it difficult to sustain a conventional TM guided mode at wavelengths near the excitonic resonances. Indeed, in contrast to TE guided modes, TM guided modes exhibit a thickness-dependent cutoff, and in this ultrathin limit the slab is not expected to support such a standard TM guided-mode branch. Therefore, the TM optical response is governed instead by the hyperbolic polaritonic branch rather than by an ordinary guided-mode mechanism.

K.3 Anisotropic slab model

We consider the layered system air/CrSBr slab/SiO_2, with $\varepsilon_{\mathrm{sup}} = 1$, $\varepsilon_{\mathrm{sub}} = 1.46^2$, and slab thickness $d = 26$

nm. The propagation direction is taken along the crystallographic b axis, denoted as x. Therefore, the reduced TM problem uses $\varepsilon_x = \varepsilon_b(\omega)$ and $\varepsilon_z = \varepsilon_c$.

For TM polarization in a diagonal anisotropic slab, the out-of-plane wavevectors are

$$k_{z0} = \sqrt{\varepsilon_{\text{sup}} k_0^2 - k_x^2}, \tag{S13.}$$

$$k_{z1} = \sqrt{\frac{\varepsilon_x}{\varepsilon_z}(\varepsilon_z k_0^2 - k_x^2)}, \tag{S14.}$$

$$k_{z2} = \sqrt{\varepsilon_{\text{sub}} k_0^2 - k_x^2}, \tag{S15.}$$

with $k_0 = \frac{\omega}{\hbar c}$. Introducing

$$Q_0 = \frac{\varepsilon_{\text{sup}}}{k_{z0}}, \qquad Q_1 = \frac{\varepsilon_x}{k_{z1}}, \qquad Q_2 = \frac{\varepsilon_{\text{sub}}}{k_{z2}}, \tag{S16.}$$

the interface reflection coefficients are

$$r_{01} = \frac{Q_1 - Q_0}{Q_1 + Q_0}, \qquad r_{12} = \frac{Q_2 - Q_1}{Q_2 + Q_1}, \qquad r_{21} = -r_{12}, \tag{S17.}$$

Finally, the slab reflection coefficient is given by

$$r_p(\omega, k_x) = \frac{r_{01} + r_{12} e^{2ik_{z1}d}}{1 - r_0 r_{21} e^{2ik_{z1}d}}, \tag{S18.}$$

in which the denominator

$$D(\omega, k_x) = 1 - r_0 r_{21} e^{2ik_{z1}d} \tag{S19.}$$

is the relevant quantity for tracking the resonant slab modes, since the mode condition is associated with minima of $|D|$.

K.4 Raw $\mathrm{Im}(r_p)$ **map and denominator-based mode map**

A first natural visualization is the raw map of $|\mathrm{Im}(r_p(\omega,\ k_x))|$. However, in practice this quantity is dominated by a broad radiative background and by strong non-resonant features near the light-cone boundaries. As a consequence, the HEP branch becomes difficult to isolate visually. This is also explicitly noted in previous work[10]: the authors point out that maxima of $\mathrm{Im}(r_p)$ are not reliable indicators of poles for *lossy modes near the light cone*.

To reveal the HEP branch more clearly, we use the denominator of the slab response. We first define $M(\omega, k_x) = \log_{10}(1/|D(\omega, k_x)|)$, and then use the quantity $M_{\text{vis}} = 1/\log_{10}(1/|D|)$ as a visualization matric for the HEP bands. The reason this works is the following. The raw map of $|\mathrm{Im}(r_p)|$ is dominated by a broad

lossy background and by strong response near the light-cone boundaries, so the weak HEP branch is largely masked. The quantity M, being constructed from the denominator of the slab reflection coefficient, is more directly connected to the pole structure of the resonant mode. However, in its raw form it still exhibits a very large dynamic range, with the strongest features dominating the contrast. Using $M_{vis} = 1/M$ compresses this contrast in a way that suppresses the broad dominant background while enhancing weaker branch-like features. As a result, the HEP band becomes much more visible, and in particular its local backbending near X^* and X^{**} can be clearly identified.

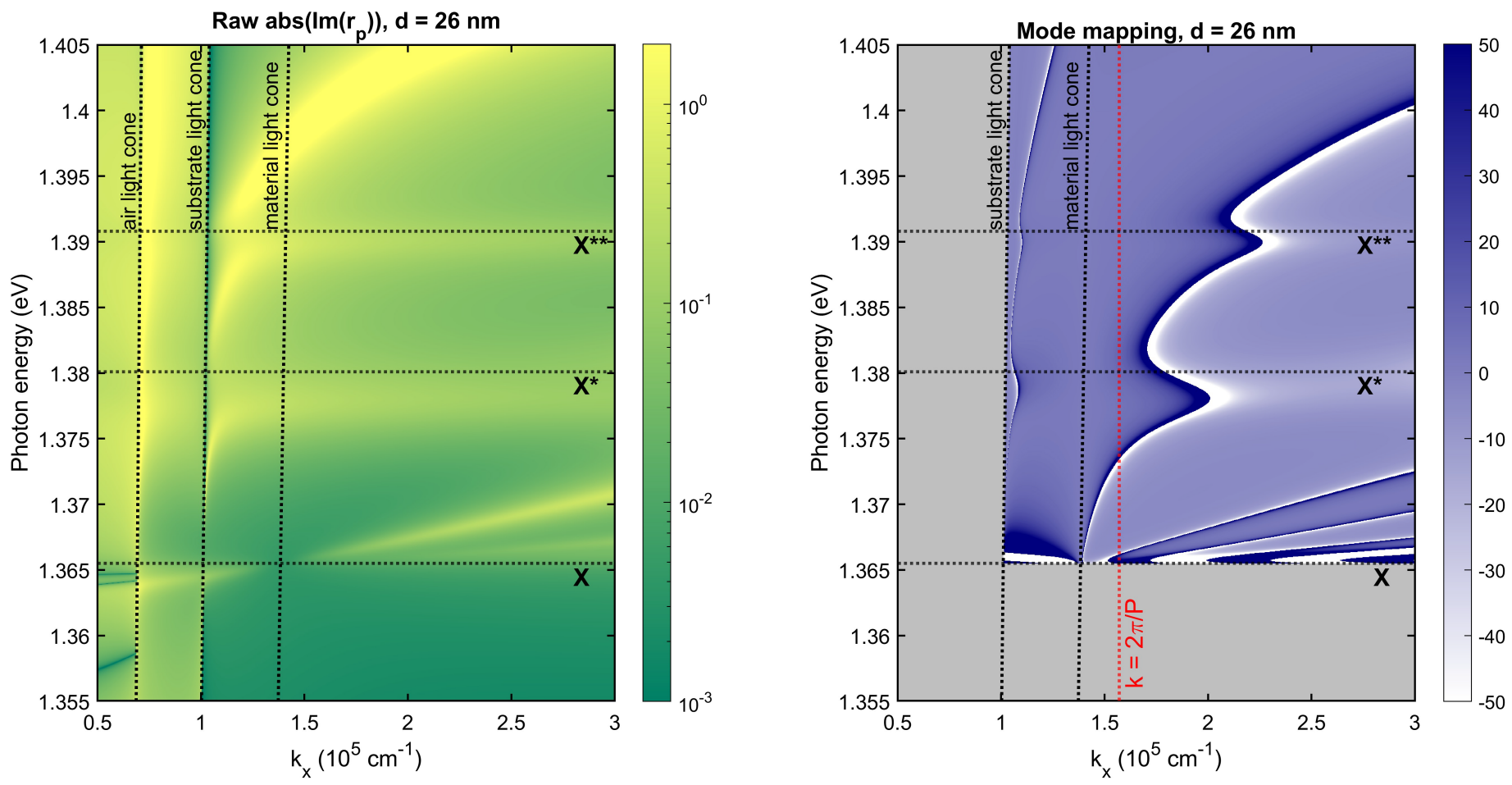

Fig. S14 | Momentum-energy maps of the patterned CrSBr slab. Left: raw $|\mathrm{Im}(r_p)|$ map, which is dominated by background response and does not reveal the HEP branch clearly. Right: denominator-based visualization using $M_{vis} = 1/\log_{10}(1/|D|)$, which makes the HEP branch visible. The shaded regions indicate the part inside the substrate light cone and the part below the main exciton energy X. These regions are excluded from the HEP branch of interest for two reasons. First, inside the substrate light cone the mode can couple efficiently to the substrate continuum and does not correspond to the deeply confined dark HEP branch targeted here. Second, below the main exciton energy X the system is outside the relevant hyperbolic excitonic window considered in this work, so the HEP branch associated with the exciton-induced hyperbolic response is not expected there. The material light cone separates the surface-HEP regime from the bulk-HEP regime.

Figure **S14** compares the raw $|\mathrm{Im}(r_p)|$ map with the denominator-based mode map. The $|\mathrm{Im}(r_p)|$ map is dominated by a broad lossy background and by strong light-cone-related response, so that it displays only smooth and relatively shallow spectral features, making the HEP branch difficult to identify unambiguously. In contrast, the denominator-based map reveals a much sharper branch-like feature, from which the HEP dispersion can be followed clearly. Most importantly, this representation resolves the local backbending of the HEP branch in the vicinity of the sideband resonances X^* and X^{**}. Such back bending is in good agreement with the behavior of HEP bands reported in the previous work[10] where similar distortions of the HEP dispersion were observed near the sideband energies.

K. 5 Surface and bulk HEPs

An important point is that the HEP can be either surface-like or bulk-like depending on its position relative to the material light cone $k_{mat} = \sqrt{\varepsilon_c} k_0$. The origin of this distinction can be understood from the out-of-plane wavevector in the anisotropic slab, $k_{z1} = \sqrt{\frac{\varepsilon_x}{\varepsilon_z}(\varepsilon_z k_0^2 - k_x^2)}$. In the hyperbolic regime relevant here, one has $\varepsilon_x < 0$ and $\varepsilon_z > 0$. Therefore, the character of k_{z1} changes when k_x crosses the material light cone.

For $k_x < k_{mat}$, one has $\varepsilon_z k_0^2 - k_x^2 > 0$. Since $\varepsilon_x/\varepsilon_z < 0$, the quantity under the square root is negative, so k_{z1} is predominantly imaginary. The field then decays evanescently along the out-of-plane direction and remains localized near the interfaces, which is why the HEP is surface-like.

By contrast, for $k_x > k_{mat}$, one has $\varepsilon_z k_0^2 - k_x^2 < 0$. In that case, the product with $\varepsilon_x/\varepsilon_z < 0$ becomes positive, so k_{z1} acquires a real oscillatory character. The field is then no longer confined, only to the interfaces but propagates across the slab thickness, corresponding to a bulk hyperbolic mode. Thus, the material light cone marks the transition from an interface-localized, surface-like HEP to a thickness-propagating, bulk-like HEP. This is in agreement with Ref[10] which identifies the fundamental HEP as surface-like inside the material light cone and bulk-like outside it, while higher- order HEPs remain bulk modes throughout the relevant momentum range.

The grating period $P = 400$ nm provides a reciprocal momentum $G = \frac{2\pi}{P} \approx 1.57 \times 10^5$ cm^{-1}, which falls in the momentum range of the first bulk HEP branch (see right image of Fig. **S14**). Therefore, as shown by the mode maps above, the mode out-coupled to the far field by our grating is naturally interpreted as the *bulk HEP band.*

References

1. Lu, L. *et al.* Engineering a light–matter strong coupling regime in perovskite-based plasmonic metasurface: quasi-bound state in the continuum and exceptional points. *Photon. Res.* **8**, A91-A100 (2020).
2. Sigurðsson, H., Nguyen, H. C. & Nguyen, H. S. Dirac exciton–polariton condensates in photonic crystal gratings. *Nanophotonics* **13**, 3503-3518 (2024).
3. Baranov, D. G. *et al.* Ultrastrong coupling between nanoparticle plasmons and cavity photons at ambient conditions. *Nat. Commun.* **11**, 2715 (2020).
4. Anantharaman, S. B. *et al.* Ultrastrong light–matter coupling in two-dimensional metal–organic chalcogenolates. *Nat. Photon.* **19**, 322-328 (2025).
5. Dirnberger, F. *et al.* Magneto-optics in a van der Waals magnet tuned by self-hybridized polaritons. *Nature* **620**, 533-537 (2023).
6. Wang, T. *et al.* Magnetically-dressed CrSBr exciton-polaritons in ultrastrong coupling regime. *Nat. Commun.* **14**, 5966 (2023).
7. Li, Q. *et al.* Two-dimensional magnetic exciton polariton with strongly coupled atomic and photonic anisotropies. *Phys. Rev. Lett.* **133**, 266901 (2024).

8 Le, N. D. *et al.* Super bound states in the continuum on a photonic flatband: concept, experimental realization, and optical trapping demonstration. *Phys. Rev. Lett.* **132**, 173802 (2024).
9 Choi, D. *et al.* Strong interlayer coupling and chiral flat-band cascades in twisted bilayer gratings. *arXiv preprint arXiv:2510.07381* (2025).
10 Ruta, F. L. *et al.* Hyperbolic exciton polaritons in a van der Waals magnet. *Nat. Commun.* **14**, 8261 (2023).
