# Supplementary material for: Observation of polaritonic flat-band bound states in the continuum in a 2D magnet
Source: Nat Commun. 2026 Aug 19;17:9908. doi: 10.1038/s41467-026-76785-w (PMC13583042; doi:10.1038/s41467-026-76785-w)
Supplement: Supplementary file 2 [file 41467_2026_76785_MOESM2_ESM.pdf]

nature portfolio

Peer Review File

Observation of polaritonic flat-band bound states in the continuum in a 2D magnet

Corresponding Author: Professor Rui Su

This file contains all reviewer reports in order by version, followed by all author rebuttals in order by version.

Version 0:

Reviewer comments:

Reviewer #1

(Remarks to the Author)
In the manuscript, the authors have reported experimental evidence of polaritonic bound state in the continuum (BIC) based on 1D nanogratings of CrSBr, a magnetic semiconductor with exceptional excitonic properties. By changing the filling factor of the grating, higher-order BICs have been observed as well, with very large Q-factors. Furthermore, when the grating directions are changed hyperbolic exciton polaritons modes also appear, with negligible angular dependence. I think these are all solid observations and should be of great interests to a broad audience. I only have a few comments that need to be addressed before the publication of this manuscript.

1. Ref. 44 also uses 1D gratings of CrSBr in the transverse electric (TE) configuration and only observed the guided mode resonance (GMR). The authors should comment on what's the difference in the current structure that enables the observation of BIC polariton. In particular, the 14 nm sample in Fig. S5 seems to have identical parameters (P=400nm, ff=0.5) as in Ref. 44.

2. The authors mentioned strong coupling/ultrastrong coupling in the CrSBr grating. What's the size of the Rabi splitting? Since an external cavity is not used, it is hard to compare with other cavity systems to assess the strong coupling/ultra strong coupling regimes. How is the coupling strength g= 118 meV compared to other cavities based on CrSBr? In Fig S2, the comparison between the model and data is displayed in a way that is hard to assess the goodness of agreement. The authors should either enlarge the bottom panels or display the results in separate figures.

3. The comparison between experiment and simulation in Fig. 2h is hard to judge. The authors should provide spectra at certain momentum to better present the comparison.

4. The opposite trend of the Q-factor with increasing momentum shown in Fig. 3g is interesting. Can that be used to distinguish GMR and BIC modes? Also, the main exciton itself already has a rather high Q-factor, with 1.36eV/0.85meV = 1600. Would BIC polariton bring any special photonic design from the BIC that can further increase the Q-factor beyond exciton's intrinsic limit?

5. "Surface hyperbolic exciton polariton" in the abstract and the main text is misleading. Surface exciton is a distinct spectral feature for CrSBr that appears at about 20 meV lower than the main (bulk) exciton of CrSBr (Nat. Mater. 24, 391 (2025)). The surface exciton's dielectric resonance is weaker than the bulk exciton in thick CrSBr and may or may not drive the dielectric function to be negative, which is a prerequisite for hyperbolicity in CrSBr. On the other hand, the hyperbolic exciton polariton (HEP) in CrSBr is usually not a surface feature (Ref. 46) since eps_c is also positive and has opposite sign compared to eps_b. Therefore, the schematic in Fig. 4a should be isofrequencies surfaces in 3D space for bulk CrSBr (26 nm). The results shown in Fig. 4c seem to be consistent with bulk HEP modes as well, where TM configurations provided momentum coupling. The authors should reconsider the terminology or provide some more evidence that the experimentally observed HEP modes are indeed confined to the surface of CrSBr.

6. Although the 2D reflectance maps are comprehensive, I think individual spectra like the ones in Fig. 4c is still very useful. The authors should consider adding some spectrum at fixed kx for the TE and TM configurations to give an overview of the GMR, BIC, and HEP modes observed in CrSBr.

Finally, the fitting parameters for the exciton and sidebands seem to be provided without a comparison between the experimental reflectance spectrum and the model fit. This should be added to the supplementary.

Reviewer #2

(Remarks to the Author)
Review of manuscript # NCOMMS-25-100789
Title: Observation of polaritonic flat-band bound states in the continuum in a 2D magnet
In their manuscript, Shen et al. investigate nanogratings fabricated in bulk van der Waals magnetic semiconductor CrSBr by angle-resolved reflectance and photoluminescence spectroscopy. They demonstrate high-order flat-band BIC with Q factors > 1500. In TM configuration, they observe that the BIC modes vanish and HEP modes emerge. The results are interesting and suitable for publication in Nat. Commun., however, several points need to be addressed before the manuscript can be accepted.
1) Overall, the language is rather poor, and the authors make several referencing errors regarding figures (listed below).
2) My impression is that the flat-band BIC claim is somewhat of an overclaim, since the LP is just very exciton-like given the large energetic detuning.
3) My impression is that the correct term here would be quasi-BIC, not BIC, since true BIC has infinite Q factor.
4) I find the use of the word “fascinating” in the abstract unusual.
5) In their introduction, the authors suggest that CrSBr is a TMDC (line 54 “unlike conventional TMDCs” and line 59 “CrSBr to stand out among other TMDCs”). This is incorrect. TMDCs are transition metal dichalcogenides. CrSBr is a transition metal chalcogen halide.
6) The authors categorize the excitons in CrSBr as main exciton and side exciton. What is a side exciton (i.e. line 146)? The excitonic resonances should be identified with reference to literature.
7) In lines 148-149, the authors refer to a “small but ambiguous red shift”. What do they mean with “ambiguous”?
8) In line 151 the authors refer to the left panel of Fig. 2d. I believe they are actually referring to the right panel.
9) In line 160, GMR and BIC should be subscripts.
10) In line 175, there is a typo “SME” should be SEM.
11) In Fig. 2g the authors use a broken y-scale. Given the data, that does not seem necessary. Why is it used?
12) In the caption of Fig 2, the authors write “(condensed) high order polaritons”. What is “condensed” supposed to mean here?
13) In the caption of Fig 2, parts f and g are incorrectly labelled as g and h.
14) The caption of Fig 4b states that it is a microscope image, which does not seem to be the case. It seems the microscope image is the inset in Fig. 4c instead.

Version 1:

Reviewer comments:

Reviewer #1

(Remarks to the Author)
In the response and revised manuscript, the authors have address most of my comments, I only have one remaining comment regarding the coupling strength.

The authors wrote: “The coupling strength g = 118 meV observed in our study is comparable to that of Fabry-Pérot (FP) cavity systems where CrSBr overlaps with the cavity modes…” Upon closer look, the comparison in Fig. S2 is made between the models and simulated angle-dependent reflectance (Fig. 1e in the main text), rather than against experimental reflectance (Fig. 2h) or PL (Fig. 2e). The authors should explain why the extraction of the coupling strength is not from comparing the USC/SC models directly with the angle-resolved experimental data. Currently, two different coupling strengths, g1=90 meV and g2=118 meV, are extracted based on calculations with fitted dielectric function, with no direct experimental constraint on the model dispersion. The authors should include experimental angle-resolved reflectance and/or PL data in Fig. S2 to better constrain the fit and the extraction of the coupling strengths.

Also, the extracted coupling strength of 118 meV in 26 nm thick CrSBr is comparable to Ref. 5 (120 meV, Nature 620, 533-537 (2023)) with a much thicker 580 nm CrSBr. The coupling strength in FP cavities scales with the square root of number of molecules/layers, so a 26 nm thick FP cavity is expected to have a coupling strength of around 25 meV. The authors attribute the larger coupling to stronger overlap between the photonic mode field and the excitonic layer in the BIC grating geometry, which is plausible but without quantitative support. The authors should provide mode-overlap calculation for the BIC structure and compare with the FP cavity, and see if the calculation is consistent with the large enhancement of the coupling strength over Ref. 5.

Reviewer #2

(Remarks to the Author)
The authors have addressed all my comments in their revised manuscript. I believe, the manuscript can bei accepted for publication in Nat. Commun. in its current form.

Version 2:

Reviewer comments:

Reviewer #1

(Remarks to the Author)
In the response and revised manuscript, the authors have addressed all my comments, I recommend the publication of the manuscript in Nature Communication in the current form.

Open Access This Peer Review File is licensed under a Creative Commons Attribution 4.0 International License, which permits use, sharing, adaptation, distribution and reproduction in any medium or format, as long as you give appropriate credit to the original author(s) and the source, provide a link to the Creative Commons license, and indicate if changes were made.
In cases where reviewers are anonymous, credit should be given to 'Anonymous Referee' and the source.
The images or other third party material in this Peer Review File are included in the article's Creative Commons license, unless indicated otherwise in a credit line to the material. If material is not included in the article's Creative Commons license and your intended use is not permitted by statutory regulation or exceeds the permitted use, you will need to obtain permission directly from the copyright holder.
To view a copy of this license, visit https://creativecommons.org/licenses/by/4.0/

Point-by-Point Responses to Reviewers' Comments

We sincerely thank all the reviewers for their constructive comments and suggestions, which allowed us to improve our manuscript. We have provided point-by-point responses to the reviewers' comments below in blue color:

Reviewer #1 (Remarks to the Author):

In the manuscript, the authors have reported experimental evidence of polaritonic bound state in the continuum (BIC) based on 1D nanogratings of CrSBr, a magnetic semiconductor with exceptional excitonic properties. By changing the filling factor of the grating, higher-order BICs have been observed as well, with very large Q-factors. Furthermore, when the grating directions are changed hyperbolic exciton polaritons modes also appear, with negligible angular dependence. I think these are all solid observations and should be of great interests to a broad audience. I only have a few comments that need to be addressed before the publication of this manuscript.

Response: We appreciate your recognition of the significance of our work. It's also rewarding that you find our results solid and of broad interest to the audience. We thank you for highlighting that our work is suitable for *Nature Communications*.

Comments:

1. Ref. 44 also uses 1D gratings of CrSBr in the transverse electric (TE) configuration and only observed the guided mode resonance (GMR). The authors should comment on what's the difference in the current structure that enables the observation of BIC polariton. In particular, the 14 nm sample in Fig. S5 seems to have identical parameters (P=400nm, ff=0.5) as in Ref. 44.

Response: We thank you for this insightful comment. In fact, the 1D photonic crystal structure (i.e., grating) adopted in our work, is similar to the structure shown in Fig. **2d** of Ref. 44 [*Nat. Photon.* **19**, 1006–1012 (2025)] in our original manuscript.

However, according to our calculations, the current structure even with slight differences in geometric parameters would not result in the absence of the BIC-polaritons. As shown in Figure **R1**, the signatures of BIC-polaritons under TE configuration are robust against variations in geometric parameters, such as thickness, period, and filling factor. This is also well-confirmed by our experimental data (e.g., Fig. **1d** and Fig. **2d** in our main text with different filling factors, and Supplementary Fig. **S5** with different thickness). In fact, it is not clear to us why BICs are absent in Ref. 44 [*Nat. Photon.* **19**, 1006–1012 (2025)] which was cited in the original manuscript. Experimentally, one possible reason is that only the spectra under normal incidence are provided that directly prohibits the observation of BICs, as BICs are intrinsically optically dark due to symmetry protection at normal incidence. In our study, we have direct experimental

access to angle-resolved spectra under a wide angular range. At finite in-plane momenta, the BIC evolves into a radiative quasi-BIC that becomes experimentally observable, which provides the direct evidence of BICs in our work. Our theoretical simulations and experimental data unambiguously confirmed the existence of BIC polaritons in CrSBr photonic crystals.

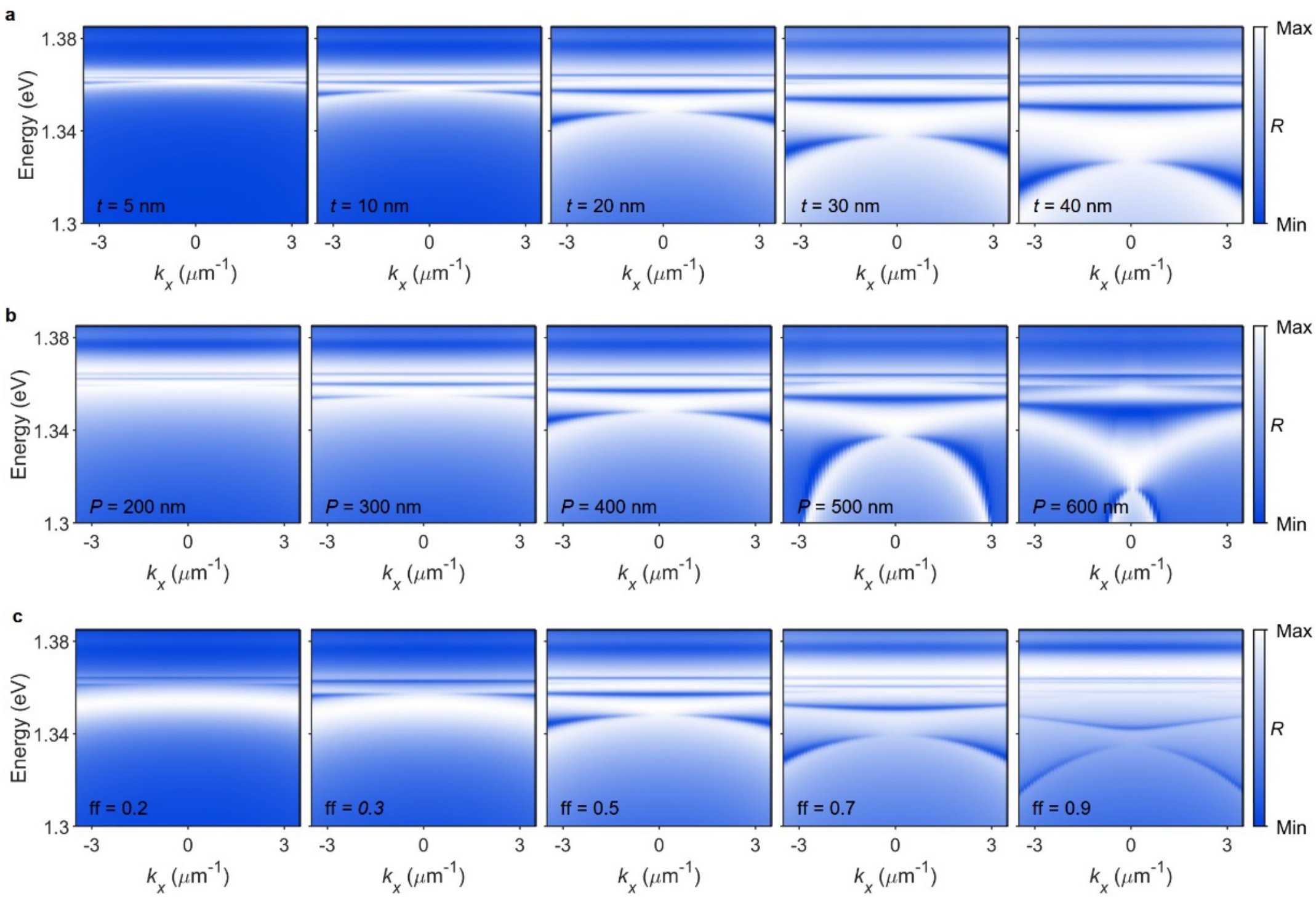

Figure **R1**. Evolution of GMR and BIC polaritons with geometric parameters. This figure is directly cited from Figure **S3** in Supplementary Materials.

2. The authors mentioned strong coupling/ultrastrong coupling in the CrSBr grating. What's the size of the Rabi splitting? Since an external cavity is not used, it is hard to compare with other cavity systems to assess the strong coupling/ultrastrong coupling regimes. How is the coupling strength g= 118 meV compared to other cavities based on CrSBr? In Fig S2, the comparison between the model and data is displayed in a way that is hard to assess the goodness of agreement. The authors should either enlarge the bottom panels or display the results in separate figures.

Response: We thank you for the useful comments to improve our work. The Rabi splitting Ω, defined as the energy difference between upper and lower polaritons at zero detuning, can be calculated using the formula $\Omega = \sqrt{4g^2 - (\gamma_c - \gamma_e)^2}$ (e.g., as indicated in Ref. [*Nat. Mater.* **22**, 970–976 (2023).]), where γ_c (γ_e) is the intrinsic loss of uncoupled photonic (excitonic) mode. Given that coupling strength is significantly larger than both γ_c and γ_e, Rabi splitting can be approximated as $\Omega \approx 2g = 236$ meV, which substantially exceeds the required criterion for the strong coupling, i.e., $[\Omega/(\gamma_c + \gamma_e)] \gg 1$. This value is also close to the

Rabi splitting of around 240 meV reported for self-hybridized polaritons based on a thick CrSBr flake (Ref. [*Nature* **620**, 533–537 (2023)]).

The comparison of the coupling strength from our work with those reported for other cavity systems using CrSBr is presented in Table **R1**. The coupling strength g= 118 meV achieved in our work is comparable to that observed in the FP cavity systems based on the bulk CrSBr flakes (for examples, in Ref. 5 [*Nature* **620**, 533–537 (2023)] and Ref. 6 [*Nat Commun* **14**, 5966 (2023)]), and larger than that of the CrSBr-photonic crystal hybrid system (Ref. 7 [*Phys. Rev. Lett.* **133**, 266901, 2024]). The variation in coupling strength can be attributed to several factors, including (i) differences in sample thickness, (ii) field confinement by different photonic modes, and (iii) the overlap of the photonic field with the excitons. The self-hybridization mechanism in our study significantly enhances the overlap between the intrinsic excitons and the mode field within CrSBr, leading to a much larger coupling strength compared to that in Ref. 7 [*Phys. Rev. Lett.* **133**, 266901, 2024], where the excitons in CrSBr couple with the evanescent field at the surface of the external PC.

Table **R1** has now been added in the revised Supplementary Materials as Table **S1**. In addition, the comparison of coupling strength between our work and other works has been added in Supplementary Materials correspondingly:

"Additionally, Table S1 presents a comparison of coupling strengths between our work and other cavity systems utilizing CrSBr. The coupling strength g = 118 meV observed in our study is comparable to that of Fabry-Pérot (FP) cavity systems where CrSBr overlaps with the cavity modes, and exceeds that found in CrSBr-photonic crystal (PC) hybrid systems employing an external PC. The variations in coupling strength between our work and others can be attributed to several factors: (i) differences in the thickness of the CrSBr samples, (ii) varying degrees of field confinement due to different optical modes, and (iii) the extent of overlap between CrSBr and the optical modes."

Refs	Cavity types	Thickness	Coupling strength
Ref. 5	Self-hybridized Fabry-Pérot cavity	~ 580 nm	~120 meV
Ref. 6	Fabry-Pérot cavity	~ 462 nm	~169 meV
Ref. 7	GMR (external	~ 6 nm	~ 58 meV

	Si_3N_4 PC)		
Our work	Self-hybridized BICs	~ 26 nm	~118 meV

Table **R1**. Comparison of coupling strengths between our work and previous works based on CrSBr.

We also thank you for the suggestions to improve the visibility of Figure **S2** in Supplementary Materials. We have enlarged the bottom panels in Figure **S2** to show a clearer comparison between models and data, as indicated in Figure **R2**.

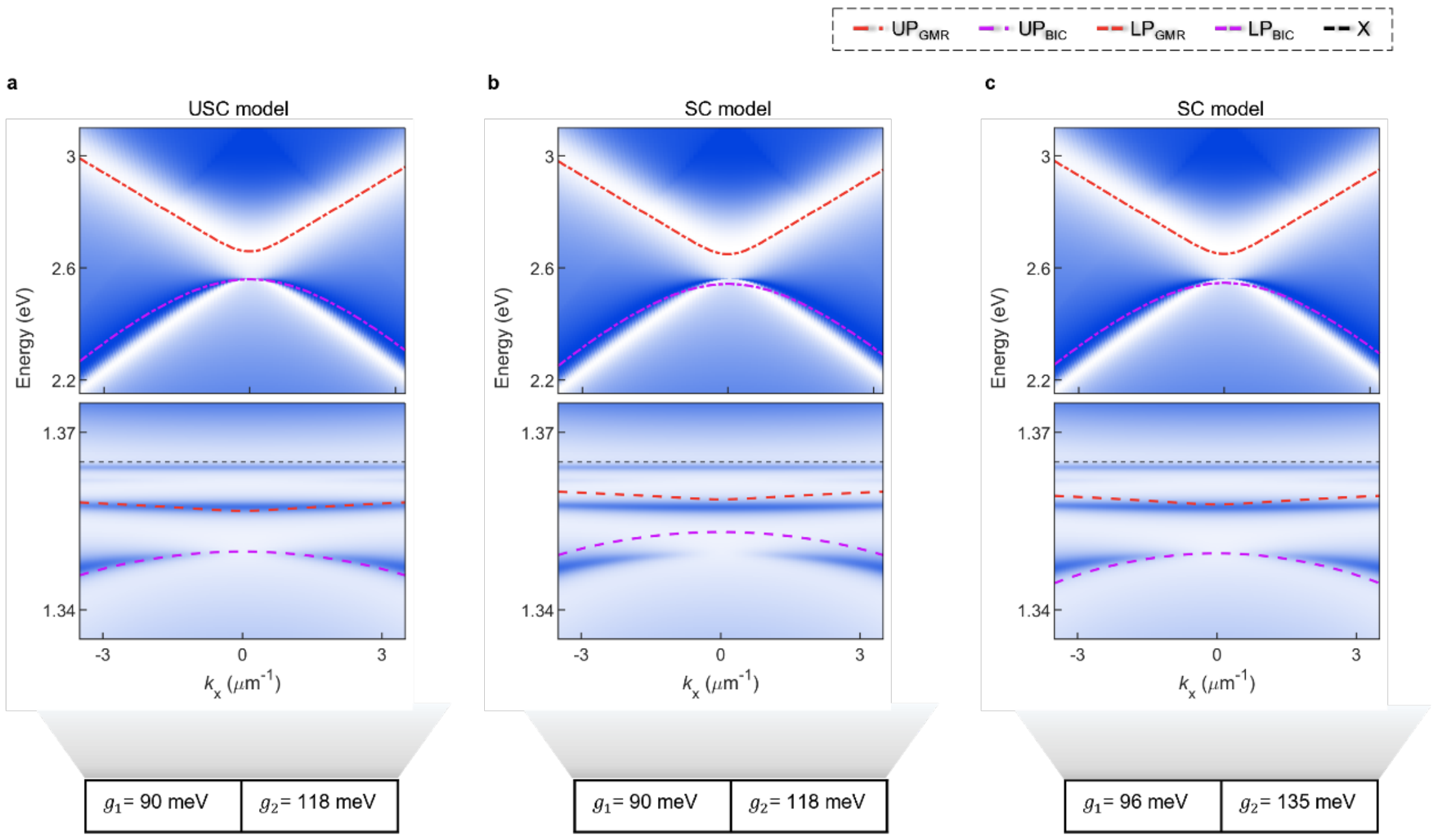

Figure **R2**. Revised Figure **S2** with enlarged bottom panels.

3. The comparison between experiment and simulation in Fig. 2h is hard to judge. The authors should provide spectra at certain momentum to better present the comparison.

Response: We thank you for the valuable comments to improve the quality of our work. To facilitate a better comparison between the simulated and measured results, in the revised manuscript we supplemented the separate reflectance spectra at k_x =0 μm^{-1} and k_x =1.8 μm^{-1}, respectively, in Figure **2i**, as also indicated in Figure **R3**. In addition, different behaviors of GMR- and BIC-polaritons in the extracted reflectance spectra at different k_x values have also been analyzed in the revised manuscript (page 5):

"We observe that the resonant frequency and linewidth of LP_{GMR} and LP_{BIC} bands remain

nearly invariant for different k_y values, which is more clearly reflected in the extracted spectra (Fig. 2i) at k_x=0 and 1.8 μm^{-1}, respectively. Moreover, the optical response vanishes at k_x=0 μm^{-1} in the LP_{BIC} band (Fig. 2i), which serves as a hallmark of BICs"

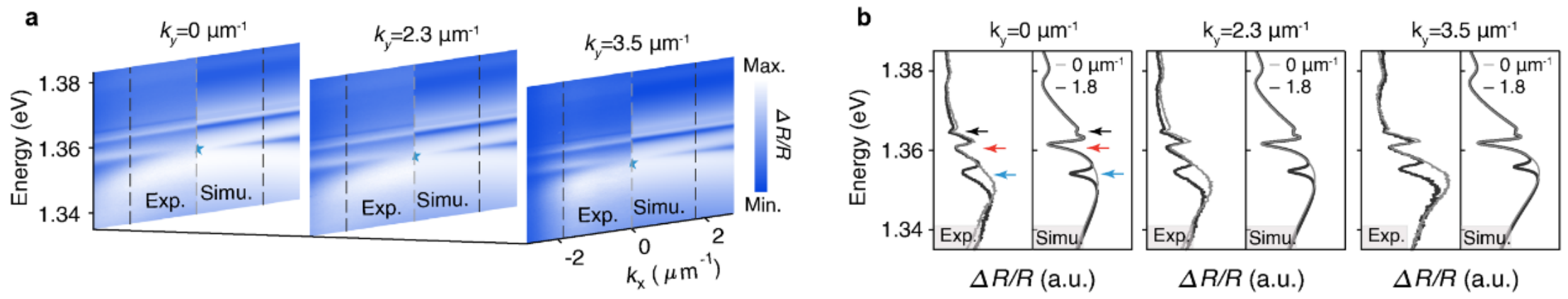

Figure **R3**. Modified Figure **2h-2i** of the revised manuscript. (**a**) Angle-resolved (along the k_x direction) reflectance spectra of CrSBr grating with k_y varied from 0, 2.3, to 3.5 μm^{-1}, respectively. Gray and black dashed lines indicate the positions at $k_x =$ 0 and 1.8 μm^{-1}, respectively. (**b**) Corresponding spectra for measured and simulated resu lts in (**a**) at k_x =0 μm^{-1} (gray curves) and k_x =1.8 μm^{-1} (black curves), respectively.

4. The opposite trend of the Q-factor with increasing momentum shown in Fig. 3g is interesting. Can that be used to distinguish GMR and BIC modes? Also, the main exciton itself already has a rather high Q-factor, with 1.36eV/0.85meV = 1600. Would BIC polariton bring any special photonic design from the BIC that can further increase the Q-factor beyond exciton's intrinsic limit?

Response: We thank you for the insightful comments to improve the quality of our work. We fully agree that the opposite trends in Q factors with increasing momentum in BIC and GMR modes are distinctive characteristics of these two modes.

This opposite trend in Q factors of GMR and BIC modes can be understood through their respective eigen-energies, given by:

$$U_{\pm} = E_m + i\gamma_r \pm \sqrt{(v_g k_x)^2 + (\xi - i\gamma_r)^2}$$

Here, U_+(BIC) and U_-(GMR) are respective eigen-energies of BIC and GMR modes. The imaginary part $\gamma_{\pm} = \mathrm{Im}[U_{\pm}]$ represents their respective radiative losses. Notably, the sum of $\gamma_{\pm}$ is conserved:

$$\gamma_+ + \gamma_- = \mathrm{Im}[U_+] + \mathrm{Im}[U_-] = \mathrm{Im}[U_+ + U_-] = 2\gamma_r.$$

For BIC modes, $\gamma_+ = 0$ at $k_x = 0$ μm^{-1}, corresponding to an infinite Q factor of true BICs at the Γ point. As k_x increases, BICs evolve into quasi-BICs, with corresponding increases in γ_+ due to enhanced asymmetry, which consequently leads to a decrease in the Q factors. Conversely, for the GMR mode, $\gamma_- = 2\gamma_r$ reaches its maximum at $k_x = 0$ and gradually

decreases as k_x increases, leading to the opposite trend of Q factors for the GMR mode compared to the BIC mode.

We have highlighted the opposite trends of Q factors for BIC- and GMR-polaritons in the revised manuscript (page 7):

"Regarding the Q factors, similar to the first-order modes, high-order LP_{BIC} and LP_{GMR} bands exhibit opposite trends with the increasing k_x (Supplementary Section A)."

Moreover, in the revised Supplementary Materials (page 3), we have added the discussion of the evolution of Q factors for GMR- and BIC-bands, based on the analysis on their respective eigen-energies:

"Notably, the sum of $\gamma_\pm$ is conserved: $\gamma_+ + \gamma_- = \mathrm{Im}[U_+] + \mathrm{Im}[U_-] = \mathrm{Im}[U_+ + U_-] = 2\gamma_r$. For BIC modes, $\gamma_+ = 0$ at $k_x = 0$ μm^{-1}, corresponding to an infinite Q factor of true BICs at the Γ point. As k_x increases, BICs evolve into quasi-BICs, with corresponding increases in γ_+ due to enhanced asymmetry, which consequently leads to a decrease in the Q factors. Conversely, for the GMR mode, $\gamma_- = 2\gamma_r$ reaches its maximum at $k_x = 0$ and gradually decreases as k_x increases, leading to the opposite trend of Q factors for the GMR mode compared to the BIC mode."

Regarding the Q factor or linewidth, polaritons are hybrid exciton-photon quasiparticles and the polariton linewidth comes from both the contributions from the exciton and photon components. As the Q factor of pure photonic BICs can reach infinity that is much larger than the exciton Q factor, it would be possible for the total Q factor (Q_{tot}) of LP_{BIC} to surpass the intrinsic limit of excitons if the contribution of BICs becomes large to the resulting polaritons. To go beyond the intrinsic limits of exciton, the contribution of BICs to lower polariton should be increased by reducing the detuning between photonic BICs and exciton energy. This can be accomplished by increasing the filling factor or the grating period (as indicated in Figure **R1**) to facilitate a redshift of the BIC mode.

In the revised manuscript (page 7), we have added the discussion of possibilities to enhanced Q factor of LP to go beyond the limits set by the intrinsic excitons:

"Increasing the contributions of photonic BIC-band to LPs, for example, by increasing the period or filling factor of gratings, is expected to further increase the total Q factor beyond the intrinsic loss by exciton."

5. "Surface hyperbolic exciton polariton" in the abstract and the main text is misleading. Surface exciton is a distinct spectral feature for CrSBr that appears at about 20 meV lower than the main (bulk) exciton of CrSBr (Nat. Mater. 24, 391 (2025)). The surface exciton's dielectric resonance is weaker than the bulk exciton in thick CrSBr and may or may not drive the dielectric function to be negative, which is a prerequisite for hyperbolicity in CrSBr. On the other hand,

the hyperbolic exciton polariton (HEP) in CrSBr is usually not a surface feature (Ref. 46) since eps_c is also positive and has opposite sign compared to eps_b. Therefore, the schematic in Fig. 4a should be isofrequencies surfaces in 3D space for bulk CrSBr (26 nm). The results shown in Fig. 4c seem to be consistent with bulk HEP modes as well, where TM configurations provided momentum coupling. The authors should reconsider the terminology or provide some more evidence that the experimentally observed HEP modes are indeed confined to the surface of CrSBr.

Response: We thank you for the useful comments and suggestions to improve our work. The terminology "surface HEPs" was initially adopted to indicate that the field distributions (as indicated in Fig. **4c** of the main text) of HEPs are predominantly confined at the interface between CrSBr and its surroundings. This is in stark contrast to GMR- or BIC-polaritons under the TE configuration, where the mode field is primarily confined within the inner part (Fig. **1f** and Fig. **3b** in the main text) of the CrSBr flake. We apologize for the confusion and did not intend to mean that hyperbolic exciton polariton comes from the surface excitons.

Indeed, we agree that exciton X at approximately 1.3655 eV in our 26 nm CrSBr sample corresponds to the bulk exciton, as the resonance energy aligns with the value reported for bulk excitons (Ref. [Nat. Mater. 24, 391–398 (2025)]). Moreover, the bulk exciton is expected to dominate for the thickness up to 26 nm.

Additionally, we also acknowledge that HEP modes observed in our study are consistent with bulk HEP modes reported in Ref. [Nat Commun 14, 8261 (2023)], where TM configurations provided momentum coupling. The reciprocal lattice vector for grating structure with period P = 400nm can be calculated as G=$(2\pi/P)$ ≈1.57×10^5 cm^{-1}, which falls in the momentum range of bulk HEPs reported in Ref. [Nat Commun 14, 8261 (2023)]. We have also calculated the HEP branches for a 26-nm CrSBr slab, as shown in Figure **R4**. The results clearly show that the reciprocal lattice vector G falls within the bulk HEP range.

To avoid misunderstandings, the terminology "HEP" instead of "surface HEP" has been adopted in the revised manuscript. In the revised Supplementary Materials, we have added the discussion of the HEPs in Supplementary Section **K** and included Figure **R4** in Supplementary Figure **S12,** highlighting that the HEPs in our CrSBr grating correspond to the bulk HEPs. In addition, we have also emphasized in the revised manuscript that the HEPs observed in our study arise from the bulk HEPs that are coupled out into the free space by the TM configurations of CrSBr gratings (page 8):

"The period P=400 nm (G~15.7 μm^{-1}) is chosen to match the momentum range of bulk HEP which is beyond the light cone of CrSBr (more details can be seen in Supplementary Section K)."

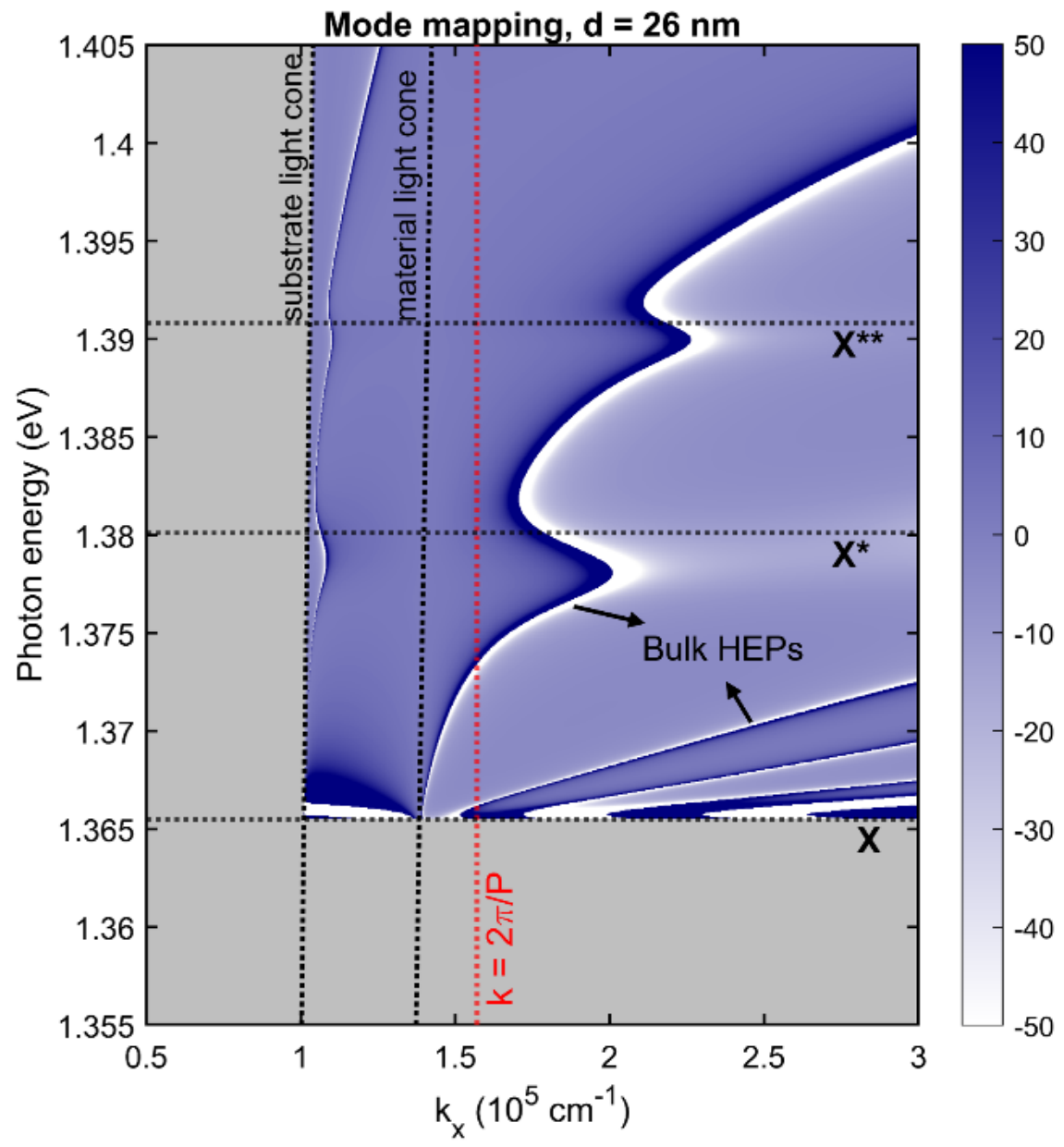

Figure R4. Mode mapping to show the HEP branches by a 26 nm-thick CrSBr slab. The shaded regions indicate the part inside the substrate light cone and the part below the main exciton energy X. These regions are excluded from the HEP branch of interest for two reasons. First, inside the substrate light cone the mode can couple efficiently to the substrate continuum and does not correspond to the deeply confined dark HEP branch targeted here. Second, below the main exciton energy X the system is outside the relevant hyperbolic excitonic window considered in this work, so the HEP branch associated with the exciton-induced hyperbolic response is not expected there. The material light cone separates the surface-HEP regime from the bulk-HEP regime.

Following the suggestion, we have calculated the 3D isofrequency surfaces (Figure **R5**) for CrSBr based on its anisotropic dielectric tensor (similar to Fig. 2b in Ref. [*Nat Commun* **15**, 69 (2024).]), which have been included in Figure **4a** of the revised manuscript.

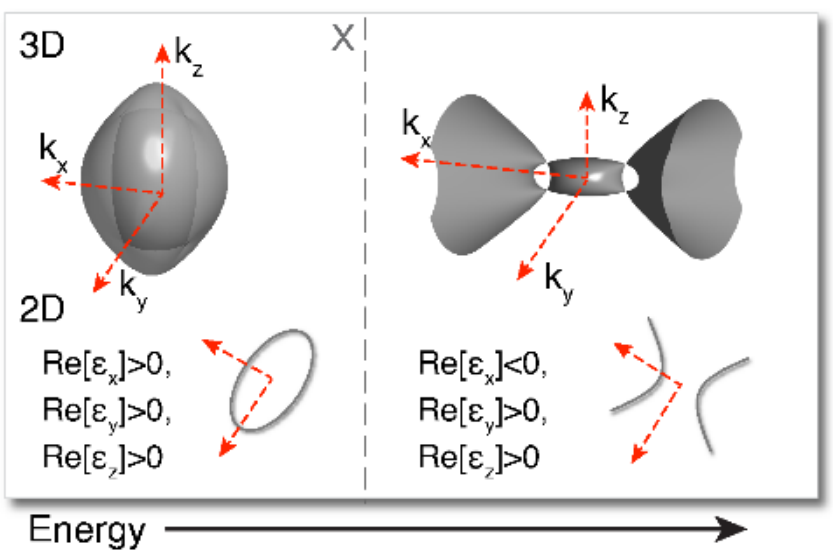

Figure **R5**. Schematic of the isofrequency surface of CrSBr at energy below (left panel) and above (right) the excitonic resonance. The corresponding 2D isofrequency contours are shown below.

Correspondingly, the 3D isofrequency surface for CrSBr at different regions has been discussed in the revised manuscript (page 7):

"Below the energy of exciton X, CrSBr is a usual elliptical material (i.e., Re[ε_x]≠Re[ε_y]≠Re[ε_z] > 0, here *x*-, *y*-, and *z*-axes correspond to b-, a-, and c-axes, respectively, as indicated in Fig. 4b) whose isofrequency surface consists of two closed surfaces (left part in Fig. 4a). This scenario corresponds to the above discussion under TE configuration. However, above the energy of exciton resonance, the large oscillator strength and the sharpened linewidth of exciton X drive the permittivity to be negative along the b-axis, yielding excitonic Reststrahlen (RS) band[46]. Within this band, the isofrequency surface becomes highly nontrivial as shown in the right panel of Figure 4a. An isofrequency surface with Re[ε_y] ≠Re[ε_z] > 0 > Re[ε_x] consists of a hyperboloid opening along the k_x direction[47], thus enabling the generation of hyperbolic exciton-polaritons (HEPs)[39]."

Additionally, the equation for 3D isofrequency surface was included in the **Method** section in the revised manuscript (page 10):

"For plane waves traveling inside an anisotropic medium, the wave equation can be given by[49]

$$\boldsymbol{k} \times (\boldsymbol{k} \times \boldsymbol{E}) + k_0^2 \boldsymbol{\varepsilon} \boldsymbol{E} = 0, \quad (3)$$

where *E* is the electric field, and *k* and k_0 are the wavevectors in the medium and free space, respectively, and ε is the permittivity tensor. By applying the condition for the nontrivial plane wave solution in eq (3), one can obtain

$$\begin{bmatrix} \varepsilon_{xx}k_0^2 - k_x^2 - k_y^2 & k_x k_y & k_z k_x \\ k_x k_y & \varepsilon_{yy}k_0^2 - k_z^2 - k_x^2 & k_y k_z \\ k_z k_x & k_y k_z & \varepsilon_{zz}k_0^2 - k_x^2 - k_y^2 \end{bmatrix} = 0, \quad (4)$$

where ε_{xx} , ε_{yy} , and ε_{zz} are the x-, y-, and z-components of dielectric tensor, corresponding to ε_a, ε_b, ε_c. The allowed *k* vectors can be c alculated by solving eq (4), giving the isofrequency surface at fixed energy.

6. Although the 2D reflectance maps are comprehensive, I think individual spectra like the ones in Fig. 4c is still very useful. The authors should consider adding some spectrum at fixed kx for the TE and TM configurations to give an overview of the GMR, BIC, and HEP modes observed in CrSBr.

<u>**Response:**</u> We thank you for the useful suggestions to improve our work. In addition to the updated Figure **2h-2i**, we have also added the spectra at fixed k_x for <u>high-order GMR- and BIC-polaritons</u> in the updated Figure **3d-g**, as indicated in Figure **R6** bel

ow. Furthermore, we have added Figure **S9** (as indicated in Fig. **R7**) in the revised Supplementary Materials to exhibit these high-order modes <u>for different k_y values</u>. Additionally, <u>for HEPs</u>, we have included Figure **S10** in the revised Supplementary Materials to exhibit the reflectance and PL spectra at some specific k_x, as shown in Figure **R8**.

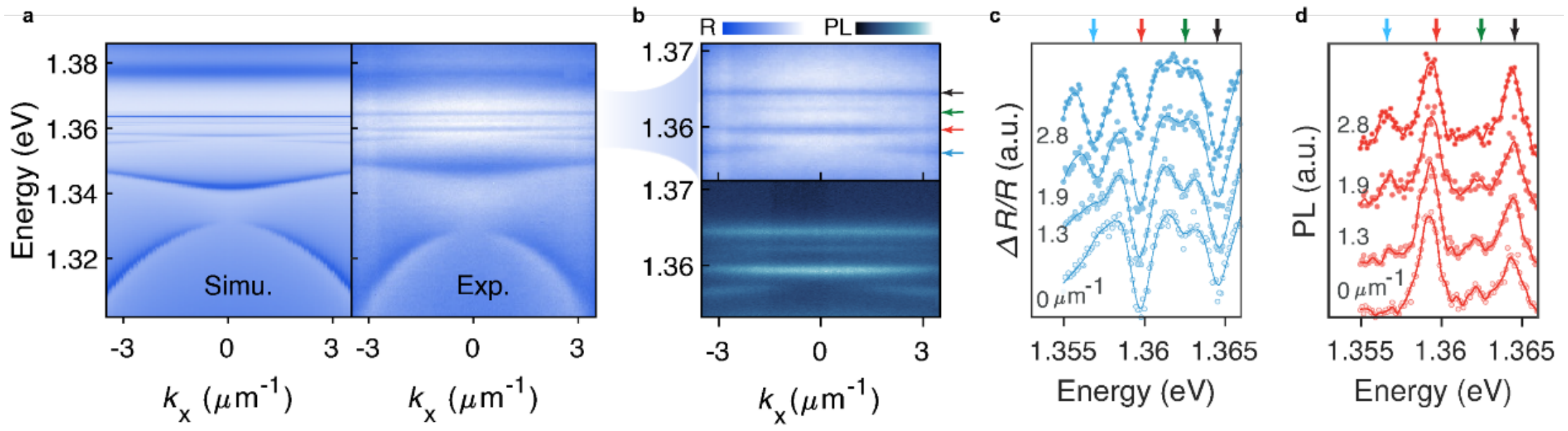

Figure **R6**. Updated Figure **3d-3g** in the revised manuscript. Reflectance (**c**) and PL (**d**) spectra at k_x =0, 1.3, 1.9, and 2.8 μm^{-1} were supplemented. Blue, red, green, and black arrows indicate the positions of second-order BIC, second-order GMR, third-order GMR, and assembly of high-order modes, as indicated in the manuscript.

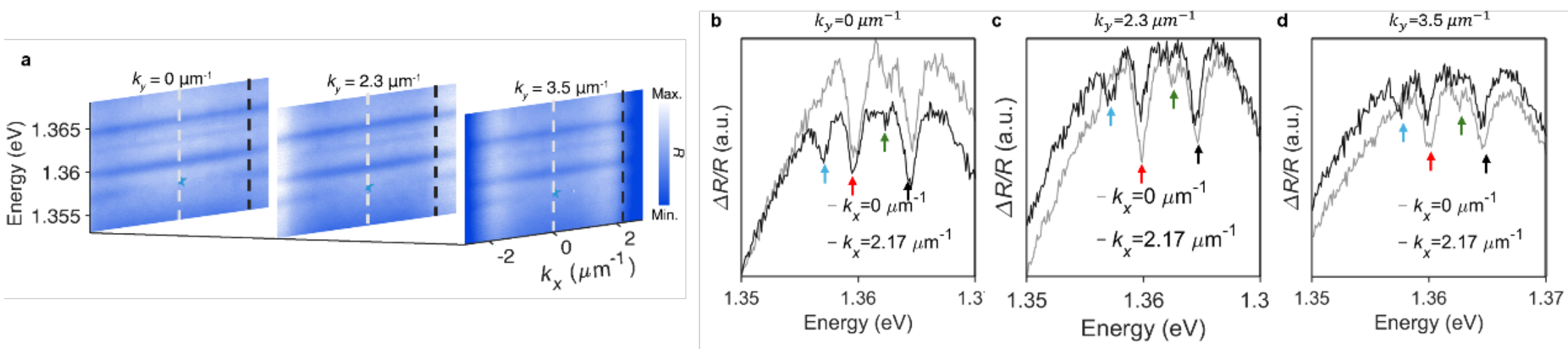

Figure **R7**. Supplemented Figure **S9** in the revised Supplementary Materials. (**a**) Angle-resolved reflectance spectra for high-order polaritonic GMR- and BIC-bands at various k_y values. Gray and black dashed lines indicate the positions at $k_x = 0$ and 2.17 μm^{-1}, respectively. (**b-d**) Extracted spectra at fixed k_x=0 and 2.17 μm^{-1}, corresponding to the data presented in (**a**).

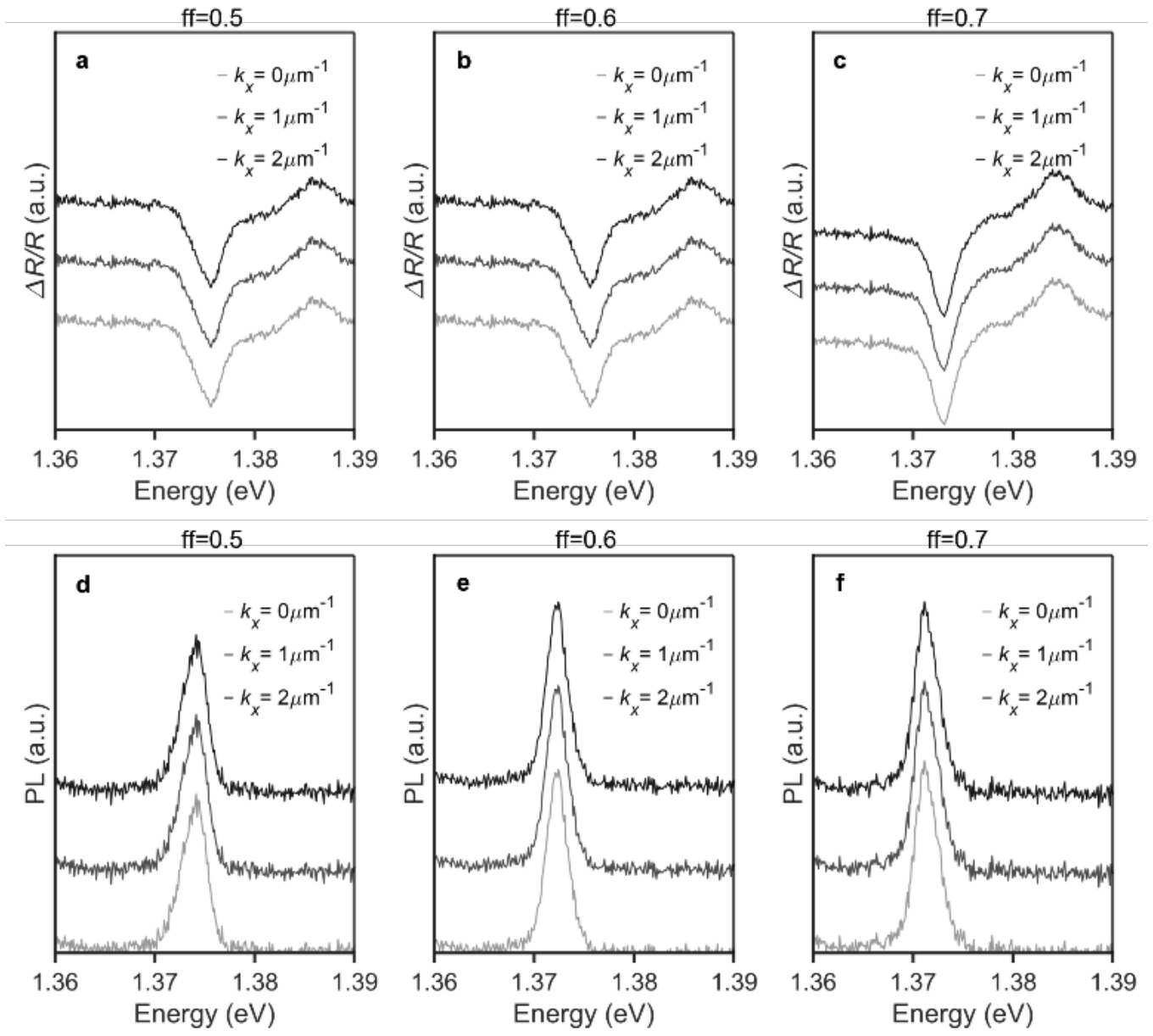

Figure **R8.** Supplemented Figure **S10** in the revised Supplementary Materials. (**a-c**) Reflectance spectra of HEP modes at k_x =0, 1, and 2 μm^{-1} for filling factor of 0.5 (**a**), 0.6 (**b**), and 0.7 (**c**), respectively. (**d-f**) PL spectra of HEP modes at k_x =0, 1, and 2 μm^{-1} for filling factor of 0.5 (**d**), 0.6 (**e**), and 0.7 (**f**), respectively.

Finally, the fitting parameters for the exciton and sidebands seem to be provided without a comparison between the experimental reflectance spectrum and the model fit. This should be added to the supplementary.

Response: We appreciate your insightful comments, which have enhanced the quality of our work. The comparison between the experimental reflectance spectrum and the model fit has now been included in the revised Supplementary Materials as Figure **S11**, as indicated in Figure **R9** below.

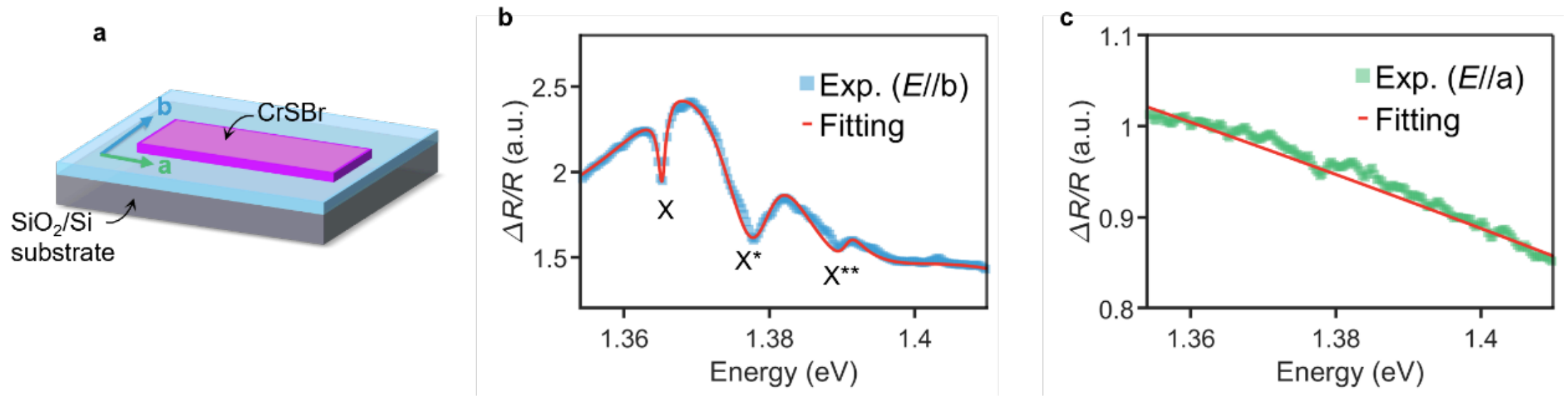

Figure **R9**. (**a**) Schematic setup of CrSBr flake on the SiO_2 (300 nm)/Si substrate. (**b**) Comparison of difference reflectance spectra (blue squares) along *b*-axis with the transfer matrix method (TMM) fitting (red curve). (**c**) Comparison of difference reflectance spectra (green squares) along *a*-axis with the TMM fitting (red curve).

Reviewer #2 (Remarks to the Author):

Review of manuscript # NCOMMS-25-100789

Title: Observation of polaritonic flat-band bound states in the continuum in a 2D magnet

In their manuscript, Shen et al. investigate nanogratings fabricated in bulk van der Waals magnetic semiconductor CrSBr by angle-resolved reflectance and photoluminescence spectroscopy. They demonstrate high-order flat-band BIC with Q factors > 1500. In TM configuration, they observe that the BIC modes vanish and HEP modes emerge. The results are interesting and suitable for publication in Nat. Commun., however, several points need to be addressed before the manuscript can be accepted.

Response: We thank you for the positive evaluation of our work and for acknowledging its significance. We appreciate your thorough review and thank you for highlighting that our work is suitable for publication in *Nature Communications*.

1) Overall, the language is rather poor, and the authors make several referencing errors regarding figures (listed below).

Response: We thank you for this important comment to improve our work. We have thoroughly refined the language in the revised manuscript and have made detailed corrections for all typos, as shown in the tracked-changes version of revised manuscript.

2) My impression is that the flat-band BIC claim is somewhat of an overclaim, since the LP is just very exciton-like given the large energetic detuning.

Response: We thank you for your insightful comment. A flat band in general describes an energy band whose energy is independent (or nearly independent) of momentum in momentum space. We agree with you that the flat band observed in the LP is primarily attributed to the dominant contributions from excitons, whose energy dispersion is constant with respect to in-plane momentum and thus results in the flat-band characteristics. In fact, in the original manuscript (page 4), we briefly indicated this excitonic-like behavior of LPs which stated that: "In contrast, the lower polaritons show significantly suppressed angular dispersion, owing to dominant excitonic weight". In our study, the achievement of flat-band LP benefits from the near ultrastrong coupling regime arising from the large excitonic oscillator strength in CrSBr. This ultrastrong coupling regime enables exciton-like behavior of LPs even at large detuning (> 1eV), while still maintaining sufficient separation to the bare exciton level.

To substantiate our claim of polaritonic flat-band BICs, we first validate the BIC nature of polaritons through the vanishing signature at the Γ point in angle-resolved reflectance and PL spectra. Additionally, we analyze the dispersion of the LP_{BIC} band, noting that it exhibits negligible shifts across the entire in-plane momentum space, in both the k_x and k_y directions.

This evidence supports the confirmation of the flat-band nature of the LP_{BIC}.

In the revised manuscript (pages 6-7), we have further highlighted the formation of the flat-band LP_{BIC} is primarily ascribed to the dominant contributions by excitons:

"The achievement of polaritonic flat bands benefits from the near-ultrastrong coupling regime arising from the large excitonic oscillator strength in CrSBr. This ultrastrong coupling regime enables exciton-like behaviour of LPs at large detuning (> 1eV), while still maintaining sufficient separation to the bare exciton level."

3) My impression is that the correct term here would be quasi-BIC, not BIC, since true BIC has infinite Q factor.

Response: We thank you for this meaningful comment. We totally agree that only the mode at k_x=0 μm^{-1} (i.e., at the Γ point) is the true BIC which feature infinite radiative Q factors (manifested as the vanishing signature at the Γ point). Deviation of normal incidence will lift the symmetry protection, rendering the true BICs evolving into quasi-BICs with finite Q factors. Consequently, the Q factors and resonance frequency shown in Figure 2f, 2g and Figure 3h, 3i are from quasi-BIC modes.

In the revised manuscript, we have highlighted that these off-Γ modes are due to the quasi-BICs (page 5, 6, and 7):

"In contrast, the UP_{BIC} band manifests as a vanishing mode (true BICs) as it approaches the normal direction (at the Γ point), evolving into quasi-BICs with finite linewidth when the symmetry protection is lifted at finite in-plane momentum. Similar to LP_{GMR}, the LP_{BIC} band shows a reduced linewidth and greatly suppressed angular dispersion compared to its upper-branch counterpart (the UP_{BIC} band)."

"In contrast, the Q factor (attributed to the quasi-BIC modes at finite in-plane momentum) of LP_{BIC} band significantly decreases from around 700 at k_x=1 μm^{-1} to approximately 400 at k_x=3 μm^{-1} due to the increased asymmetry as k_x increases." (page 5)

"Compared to the second-order LP_{GMR} band whose optical response is almost constant, the second-order LP_{BIC} band exhibits the transition from BIC mode to quasi-BIC mode when the symmetry is broken at finite k_x." (page 6)

"In contrast, quasi-BIC modes in second-order LP_{BIC} band exhibit an unambiguous decrease as k_x increases, with its largest Q factor exceeding 1500 at k_x =1.25 μm^{-1}, …" (page 7)

Correspondingly, to avoid misunderstandings, the terminologies 'photonic BIC-band' and 'polaritonic BIC-band' or 'LP_{BIC} band' have been defined in the revised manuscript. These terms refer to the photonic and polaritonic bands that sustain the BIC modes at the Γ point, with

similar definitions also applied to GMR. Especially, in our revised manuscript we have highlighted that the term 'polaritonic flat-band BICs', which is the main claim of our work, refers to the BIC-polaritons existing in a wide-angle flat band. Related statements involving these definitions from the revised manuscript are listed below (page 2 and 3):

"Here, we experimentally demonstrate high-order polaritonic BICs on a wide-angle flat band, denoted as "polaritonic flat-band BICs", based on vdW magnet CrSBr metasurfaces." (page 2)

"Correspondingly, the photonic bands sustaining these two modes will be denoted as the "photonic GMR-band" and "photonic BIC-band", respectively." (page 3)

"When excitons are included in the permittivity of CrSBr, the photonic GMR-/BIC-band independently hybridizes with the excitons, resulting in the formation of upper (UP_{GMR}/UP_{BIC}) and lower (LP_{GMR}/LP_{BIC}) polaritonic GMR-/BIC-bands …" (page 3)

4) I find the use of the word "fascinating" in the abstract unusual.

Response: We thank you for pointing out this issue. We have deleted the word "fascinating" in the revised abstract:

"In addition, we find that these polaritonic BIC-bands vanish in the transverse magnetic configuration, while leading to hyperbolic exciton-polaritons within the Reststrahlen band."

5) In their introduction, the authors suggest that CrSBr is a TMDC (line 54 "unlike conventional TMDCs" and line 59 "CrSBr to stand out among other TMDCs"). This is incorrect. TMDCs are transition metal dichalcogenides. CrSBr is a transition metal chalcogen halide.

Response: We thank you for pointing out these inaccuracies. To address your concerns, we have corrected the relevant expressions in the revised manuscript (page 2):

"Unlike TMDCs, it features anisotropic direct-bandgap excitons across all thicknesses[34-38]." (page 2)

"These unique characteristics enable the ultrastrong exciton-photon coupling regime even in a single CrSBr flake[34,35],which distinguishes CrSBr from other vdW materials, highlighting its potential to strengthen BIC–exciton coupling and enable polaritonic flat-band BICs over a broad angular range. However, the experimental demonstration remains elusive." (page 2)

6) The authors categorize the excitons in CrSBr as main exciton and side exciton. What is a side exciton (i.e. line 146)? The excitonic resonances should be identified with reference to literature.

Response: We thank you for this constructive comment. For CrSBr, in addition to the main contribution by exciton X, there are also the sidebands of the main exciton X, usually labelled as X* and X**, but with smaller oscillator strengths and broader linewidths compared to exciton X, as reported in previous literatures (e.g., Ref. [*Nat Commun* **14**, 8261 (2023)]).

In the revised manuscript, we have identified these excitons and included related references to clarify their roles (page 3):

"Additionally, we note the existence of sidebands above the energy of exciton X, labelled X* and X[39], which exhibit much lower oscillator strengths and broader linewidths compared to X (see Methods)."**

7) In lines 148-149, the authors refer to a "small but ambiguous red shift". What do they mean with "ambiguous"?

Response: We apologize for the typo and thank you for pointing it out. The corrected word "unambiguous" was used in the revised manuscript (page 5):

"Notably, an additional polaritonic band, marked by a black arrow, appears below the energy of exciton X with a small but unambiguous red shift."

8) In line 151 the authors refer to the left panel of Fig. 2d. I believe they are actually referring to the right panel.

Response: We thank you for pointing out this typo. We have corrected it in the revised manuscript (page 5):

"All of the polaritonic bands disappear when the electric field is oriented perpendicular to the b-axis (right panel of Fig. 2d), due to the quasi-1D nature of excitons."

9) In line 160, GMR and BIC should be subscripts.

Response: We thank you for pointing out this typo. We have corrected them correspondingly in the revised manuscript (page 5):

"We observe that the resonant frequency and linewidth of LP_{GMR} and LP_{BIC} bands remain nearly invariant for different k_y values, which more clearly reflected in the extracted spectra (Fig. 2i) at $k_x = 0$ and 1.8 μm^{-1}, respectively"

10) In line 175, there is a typo "SME" should be SEM.

Response: We thank you for pointing out this typo. We have corrected in the revised manuscript (page 6):

"As the filling factor further increased to ~0.72 (as the SEM image in Figure 3c shows), the first-order LP_{BIC} and LP_{GMR} bands redshift and become more angularly dispersive

(Fig. 3d).”

11) In Fig. 2g the authors use a broken y-scale. Given the data, that does not seem necessary. Why is it used?

Response: We thank you for this useful comment. The broken *y*-scale employed in Fig. **2g** was intended to differentiate different behaviors of LP_{GMR} and LP_{BIC}, as we adopted a similar way to present extracted resonance frequencies in Fig. **2f**. We agree with you that it is not necessary as the ranges of Q factors of LP_{GMR} and LP_{BIC} bands are close to each other. In the revised version, we have revised Figure **2g** using a single, continuous y-scale as shown below in Figure **R10**.

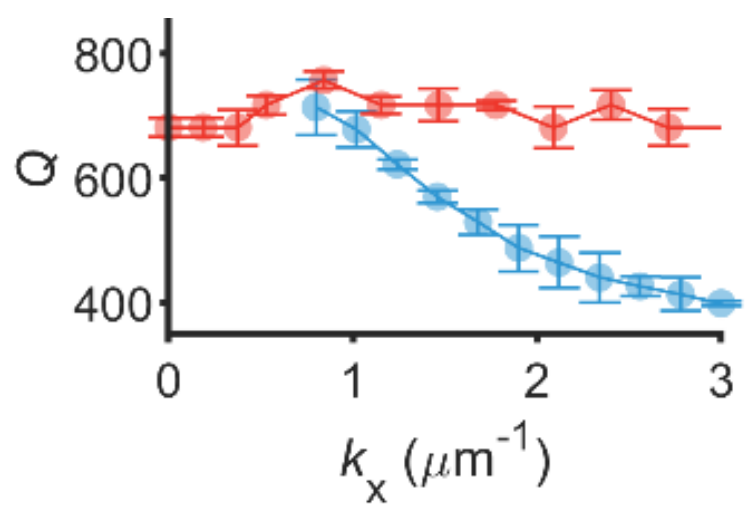

Figure **R10**. Modified image (Fig. **2g**) to show the evolution of Q factors with k_x with a single, continuous y-scale.

12) In the caption of Fig 2, the authors write “(condensed) high order polaritons”. What is “condensed” supposed to mean here?

Response: We thank you for your comment regarding the term "condensed" used in the caption of Fig. **2**. The word "condensed" was intended to describe the assembly of high-order polaritonic bands in CrSBr, as schematically illustrated in Fig. **3a** of main text. These bands are so closely spaced in energy that they cannot be distinguished in the spectra and appear as a single band. However, we recognize that this terminology would lead to confusion. In response, we have revised the caption of Figure **2** of the manuscript:

“Black and red arrows indicate the assembly of high-order polaritonic bands and the first-order LP_{GMR}, respectively.”

In addition, an illustration of this assembly of high-order polaritons has been added in the revised manuscript (page 6):

“In Figures 2d-2e, these higher-order polaritonic bands are too close in energy to be distinguished in the spectra, appearing within the same band.”

13) In the caption of Fig 2, parts f and g are incorrectly labelled as g and h.

Response: We thank you for pointing out this typo. We have corrected it in the caption of Figure

2 of the revised manuscript:

"(f-g) Evolution of resonant frequencies (f) and *Q* factors (g) as a function of k_x for LP_{GMR} (red solid circles) and LP_{BIC} (blue solid circles), extracted through Lorentzian fittings from the data in (e)."

14) The caption of Fig 4b states that it is a microscope image, which does not seem to be the case. It seems the microscope image is the inset in Fig. 4c instead.

Response: We thank you for pointing out this typo. We have corrected it in the caption of Figure **4d** (we have exchanged the labels of Fig. **4c** and Fig. **4d** in the revised manuscript to align with sequence of the content):

"(d) Normal reflectance (top panel) and PL spectra for bulk CrSBr (red curve) and gratings with varying filling factors: *ff* = 0.5 (blue curve), *ff* = 0.6 (green curve), and *ff* =0.7 (yellow curve). The slight difference between the reflectance dip and PL peak of bulk CrSBr is attributed to the Stokes shift. Inset: Microscopic image of the CrSBr gratings under the TM configuration. The scale bar represents 10 μm."

Point-by-Point Responses to Reviewers' Comments

We sincerely thank the reviewers for their constructive comments and suggestions, which allowed us to improve our manuscript. We have provided point-by-point responses to the reviewer's comments below in blue color:

Reviewer #1 (Remarks to the Author):

Comments:

1. The authors wrote: "The coupling strength g = 118 meV observed in our study is comparable to that of Fabry-Pérot (FP) cavity systems where CrSBr overlaps with the cavity modes…" Upon closer look, the comparison in Fig. S2 is made between the models and simulated angle-dependent reflectance (Fig. 1e in the main text), rather than against experimental reflectance (Fig. 2h) or PL (Fig. 2e). The authors should explain why the extraction of the coupling strength is not from comparing the USC/SC models directly with the angle-resolved experimental data. Currently, two different coupling strengths, g1=90 meV and g2=118 meV, are extracted based on calculations with fitted dielectric function, with no direct experimental constraint on the model dispersion. The authors should include experimental angle-resolved reflectance and/or PL data in Fig. S2 to better constrain the fit and the extraction of the coupling strengths.

Response: We thank you for this insightful comment for improving our work. Our angle-resolved experimental data agrees well with the simulated angle-dependent reflectance, exhibiting similar energy bands. To avoid misunderstanding, the experimental reflectance spectra (data from Fig. **2d** in the main text) together with the corresponding model fits are presented in Fig. **R1a**. The corresponding simulated reflectance spectra (data from Fig. **2h** in the main text) with the same model fits is shown in Fig. **R1b,** which align well with the experimental results. Similar coupling strength values are extracted from fitting the experimental data (g_1~85 meVand g_2~115 meV).

Figure R1 has been supplemented as Figure S3 in the revised Supplementary Materials.

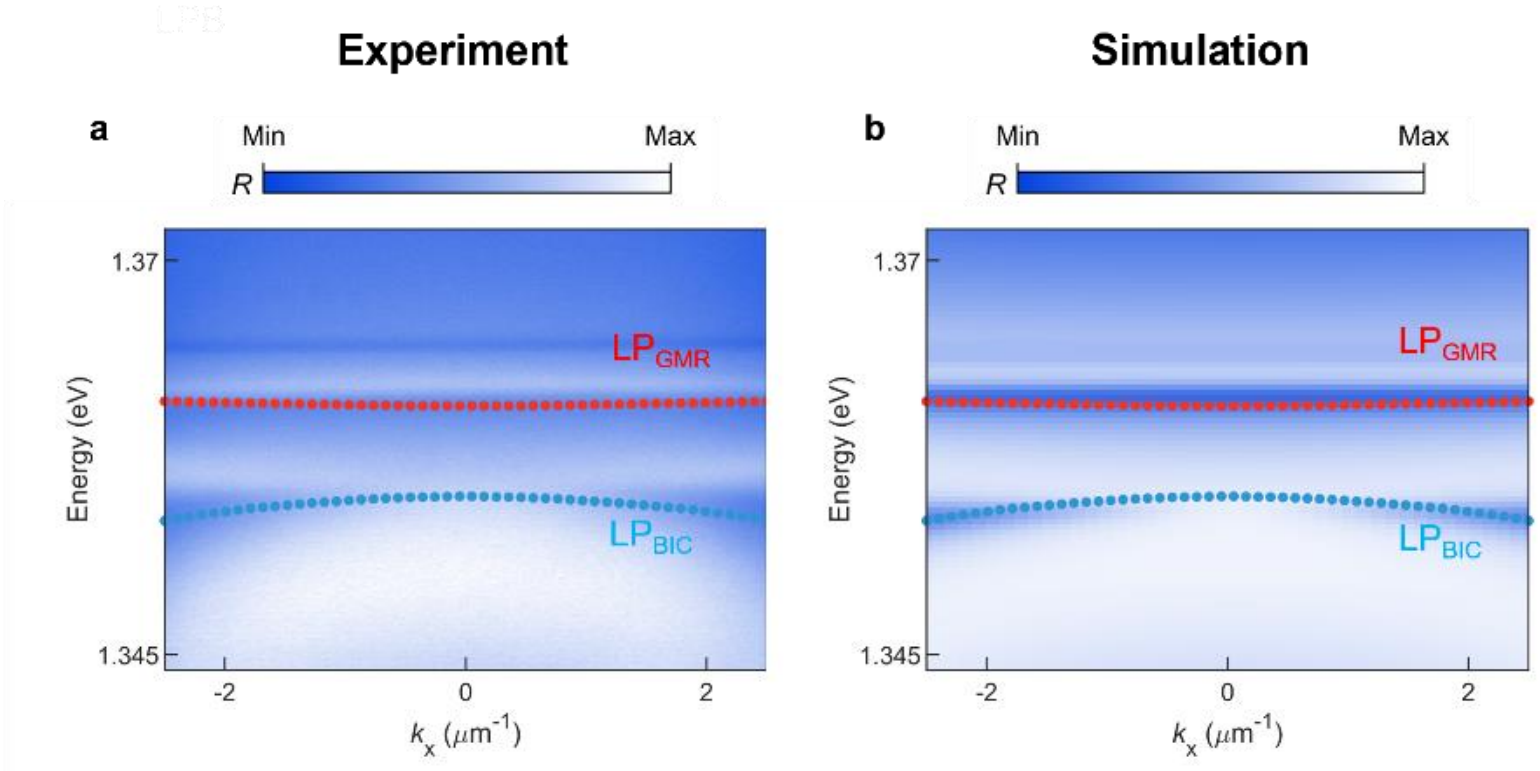

Fig. R1 | (**a**) Experimentally measured results from Figure **2d** in the main text. The red and blue dots indicate the fitted dispersions of the LP_{GMR} and LP_{BIC} branches, respectively. (**b**) Simulated reflectance spectra corresponding to (**a**).

2. Also, the extracted coupling strength of 118 meV in 26 nm thick CrSBr is comparable to Ref. 5 (120 meV, Nature 620, 533-537 (2023)) with a much thicker 580 nm CrSBr. The coupling strength in FP cavities scales with the square root of number of molecules/layers, so a 26 nm thick FP cavity is expected to have a coupling strength of around 25 meV. The authors attribute the larger coupling to stronger overlap between the photonic mode field and the excitonic layer in the BIC grating geometry, which is plausible but without quantitative support. The authors should provide mode-overlap calculation for the BIC structure and compare with the FP cavity, and see if the calculation is consistent with the large enhancement of the coupling strength over Ref. 5.

Response: We thank you for this meaningful comment. We agree with you that the coupling strength in FP cavities scales with the square root of number of molecules/layers. However, it also depends on the effective mode volume. Taken together, the coupling strength can be calculated as $g \propto \mu \cdot E \propto \sqrt{N/V}$ (see, e.g., Refs. [*Nature* **535**, 127–130 (2016), *Nat. Mater.* **22**, 970–976 (2023)]). Here, N is the exciton number, which is proportional to the geometric volume of the structure. For instance, for the grating structure (one unit) in Figure **R2a**, N is proportional to the product of width w, layer thickness t, and length l (which is infinite along the *y*-axis but can be cancelled out by V in the expression $\sqrt{N/V}$). V is the effective mode volume, which can be evaluated as

$$V = \frac{\int \varepsilon(\mathbf{r}) \mid E(\mathbf{r}) \mid^2 \, d^3\mathbf{r}}{\max[\varepsilon(\mathbf{r}) \mid E(\mathbf{r}) \mid^2]}$$

(see, e.g., Eq. (S3) in Ref. [*Nature* **535**, 127–130 (2016)] and Eq. (2) in Ref. [*Nano Lett.* **17**, 8, 4689–4697 (2017)]).

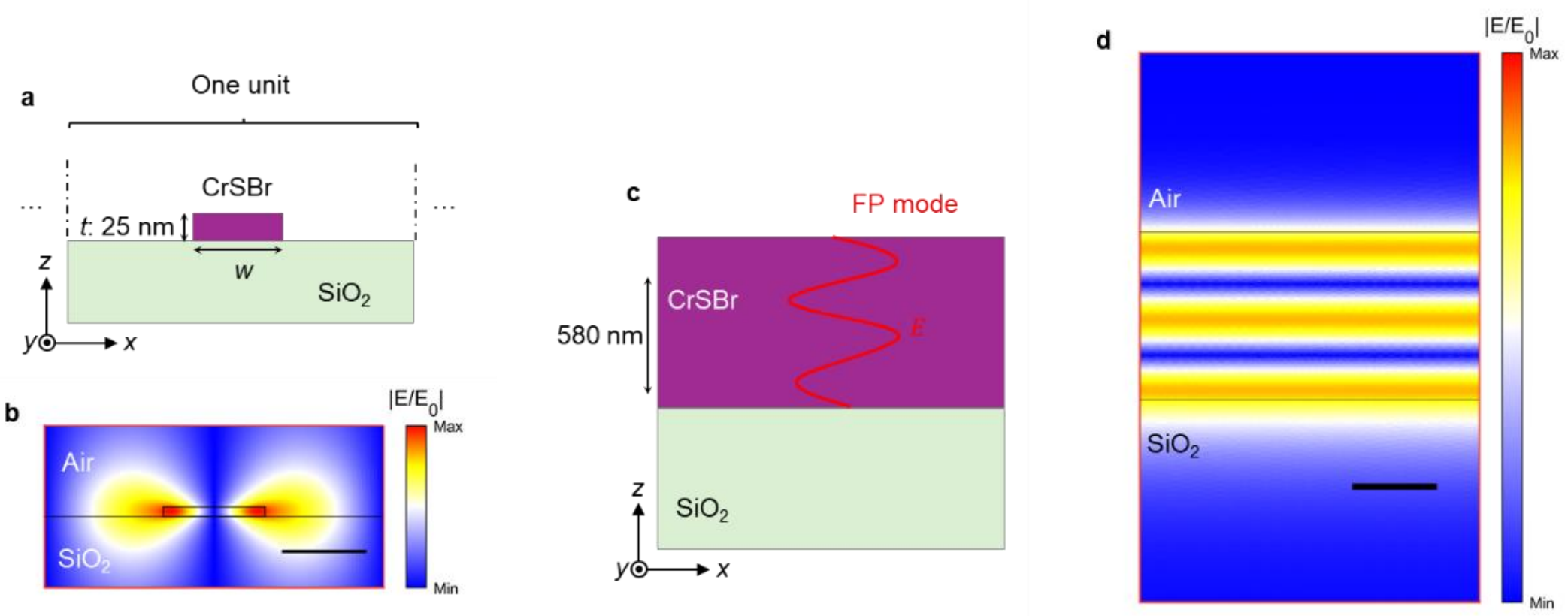

Fig. R2 | Mode distributions of the BIC and FP modes. (**a**) Schematic of the 25-nm CrSBr grating supporting a BIC mode. (**b**) Near-field distribution of the photonic BIC mode with the excitonic Lorentz oscillator removed. (**c**) Schematic of the 580-nm CrSBr flake supporting FP modes. (**d**) Near-field distribution of one photonic FP mode with the excitonic Lorentz oscillator removed.

To provide quantitative analysis of the coupling strength, we first calculated the mode distributions separately for the 25 nm–BIC structure (Fig. **R2a-b**) and the 580 nm–FP cavity (Fig. **R2c-d**), and then investigated how the coupling strengths depend on these near-field distributions. The modes were calculated using the non-resonant background dielectric tensor of CrSBr, with the excitonic Lorentz oscillator removed. This approach avoids double counting, because a simulation using the full resonant dielectric function already captures the hybrid exciton–polariton modes.

Based on the near-field distributions in Figure **R2b** and **R2d** and their respective geometric volumes, we estimate the relative coupling strengths as

$$g_{\mathrm{BIC}} \propto \sqrt{N_{\mathrm{BIC}}/V_{\mathrm{BIC}}} \approx 0.8405, g_{\mathrm{FP}} \propto \sqrt{N_{\mathrm{FP}}/V_{\mathrm{FP}}} \approx 1.31.$$

Despite the large difference in layer thickness, the coupling strength associated with the 25-nm BIC structure is close to that of the 580-nm FP cavity, yielding $g_{\mathrm{FP}}/g_{\mathrm{BIC}} \approx 1.56$.Although reducing (increasing) the thickness decreases (increases) the exciton number (i.e., N) contributing to the coupling, the effective mode volume V changes in the **same direction**. As a result, the decrease (increase) in mode volume partially **compensates** the thickness-dependent change in N, so that the net coupling strength remains comparable. The slight mismatch between the measured ratio ($\frac{g_{\mathrm{FP}}}{g_{\mathrm{BIC}}} = 120/115 \sim 1.04$) and the mode-volume estimation can be attributed to small differences in the fitted dielectric parameters used in our simulations and in Ref. [*Nature* **620**, 533–537 (2023)].

It is worth mentioning that our estimated ratio of 1.56 is consistent with some previous works in the same system. For instance, $g_{\mathrm{FP}}{\sim}169\mathrm{meV}$ and 220 meV were reported in 460-nm-thickness CrSBr (Ref. [Nat. Commun. 14, 5966 (2023)]) and 354-nm-thickness CrSBr (Ref. [*Adv. Funct. Mater.* 2024, **34**, 2411589]) self-hybridized cavities, respectively. These values give rise to respective ratios of $\frac{169}{115} \approx 1.47$ and $\frac{220}{115} \approx 1.91$, suggesting our extracted coupling strengths are reasonable.

Figure R2 (as Figure S4) and associated analyses have been added in the revised *Supplementary Materials*.
